# Targeting the Ezrin Adaptor Protein Sensitizes Metastatic Breast Cancer Cells to Chemotherapy and Reduces Neoadjuvant Therapy–induced Metastasis

**DOI:** 10.1158/2767-9764.CRC-21-0117

**Published:** 2022-06-17

**Authors:** Victoria Hoskin, Abdi Ghaffari, Brian J. Laight, Sandip SenGupta, Yolanda Madarnas, Christopher J.B. Nicol, Bruce E. Elliott, Sonal Varma, Peter A. Greer

**Affiliations:** 1 Department of Pathology and Molecular Medicine, Queen's University, Kingston, Ontario, Canada.; 2 Division of Cancer Biology and Genetics, Queen's Cancer Research Institute; Kingston, Ontario, Canada.; 3 Kingston Health Sciences Centre, Kingston, Ontario, Canada.

## Abstract

The main cause of cancer-associated deaths is the spread of cancer cells to distant organs. Despite its success in the primary tumor setting, modern chemotherapeutic strategies are rendered ineffective at treating metastatic disease, largely due to the development of resistance. The adaptor protein ezrin has been shown to promote cancer metastasis in multiple preclinical models and is associated with poor prognosis in several cancer types, including breast cancer. Ezrin promotes pro-survival signaling, particularly in disseminated cancer cells, to facilitate metastatic outgrowth. However, the role of ezrin in breast cancer chemoresistance is not fully known. In this study, we show that upregulating or downregulating ezrin expression modifies the sensitivity of breast cancer cells to doxorubicin and docetaxel treatment *in vitro* and is associated with changes in PI3K/Akt and NFκB pathway activation. In addition, we tested the effects of systemic treatment with a small-molecule ezrin inhibitor, NSC668394, on lung metastatic burden *in vivo* as a monotherapy, or in combination with anthracycline- or taxane-based chemotherapy treatment. We show that anti-ezrin treatment alone reduces metastatic burden and markedly sensitizes metastases to doxorubicin or docetaxel in neoadjuvant as well as neoadjuvant plus adjuvant treatment models. Taken together, our findings demonstrate the impact of anti-ezrin treatment in modulating response to chemotherapy in breast cancer cells as well as the efficacy of anti-ezrin treatment in combination with chemotherapy at reducing metastatic burden.

**Significance:**

This work provides preclinical evidence for combining anti-ezrin treatment with chemotherapy as a novel strategy for effectively targeting metastasis, particularly in a neoadjuvant treatment setting.

## Introduction

Chemotherapy remains one of the frontline systemic therapies for many solid tumor types and can provide long-term survival benefits for some patients. However, a subset of patients eventually develop metastasis—the main cause of cancer-related deaths—with limited available treatment options (1). Treatment options for metastatic breast cancer are limited and conventional regimens show only minimal improvements in overall survival (2). In part, this is due to the chemoresistant nature of metastatic cancer cells, arising from *de novo* drug resistance or acquired after previous chemotherapy treatment which may select for therapy-resistant cells over time (1). Furthermore, recent studies using preclinical animal models show that chemotherapy treatment, particularly in the neoadjuvant setting, can paradoxically enhance cancer cell dissemination, and may therefore compromise long-term therapeutic benefits achieved by this regimen (3, 4). For some cancers, including breast cancer, systemic spread of cancer cells may be an early event in disease progression, occurring prior to any therapeutic or surgical intervention (5). These challenges emphasize a critical need for new strategies to specifically target metastatic cancer cells, including sensitizing them to chemotherapy agents.

Ezrin, a member of the ERM (ezrin-radixin-moesin) family of cytoskeleton adaptor proteins, is frequently overexpressed in invasive cancers and is associated with disease progression and poor overall survival in multiple malignancies, including breast, pancreatic, ovarian, colorectal, and osteosarcoma (6). We recently showed ezrin to be prognostic in patients with breast cancer who are at higher risk for disease relapse (7), consistent with preclinical data from our group and others linking ezrin with a metastatic-specific function (7–12). Ezrin activity is promoted by phosphorylation at a conserved threonine residue (T-567) in the C-terminal F-actin binding domain. In the unphosphorylated state, ezrin is kept in a closed conformation by intramolecular interactions between the N-terminal FERM (four point one, ezrin, radixin, moesin) domain and the C-terminal F-actin binding domain. This masks key binding sites in the FERM domain for its plasma membrane–associated binding partners, as well as the F-actin binding domain (reviewed in ref. 13). Phosphorylation of T-567 induces conformational changes that are required for the open/active conformation of ezrin, as well as its linker function (13). As an adaptor protein, ezrin links the actin cytoskeleton to membrane proteins including CD44, ICAMs, and NHE transporters, thereby modulating signaling pathways downstream of these binding partners (14). Because of this, ezrin is implicated as a regulator of cancer metastasis, playing critical roles in cancer cell migration and invasion (7–9, 11). Ezrin is also known to regulate survival signaling (12, 15), particularly in disseminated cancer cells to facilitate metastatic seeding and outgrowth in distant organ sites (11, 12). However, it is not clear whether targeting ezrin would affect already disseminated cancer cells, or their response to chemotherapy.

In this study, we examined whether ezrin affects the sensitivity of breast cancer cells to anthracycline or taxanes using both *in vitro* and *in vivo* models. Using *in vitro* cell line models, we show that upregulating or downregulating ezrin decreased or increased, respectively, the sensitivity of breast cancer cells to doxorubicin (DOX) and docetaxel (DTX) treatment. We also demonstrate the efficacy of anti-ezrin treatment using the small molecule, NSC668394, on sensitizing micrometastases to DOX and DTX treatment *in vivo*, particularly in eliminating the increased metastatic burden induced by neoadjuvant DOX or DTX chemotherapy.

Our findings indicate that ezrin is an important modulator of chemotherapy resistance in breast cancer and that anti-ezrin therapy can sensitize metastatic breast cancer cells to DOX or DTX in preclinical models of neoadjuvant or neoadjuvant plus adjuvant treatment.

## Materials and Methods

### Cell Lines and Constructs

BT-474, MCF10A, MCF-7, T-47D, and ZR-75-1 human breast cancer cell lines were acquired from ATCC and were a gift from Dr. Christopher Mueller (Queen's University, Ontario, Canada), and they were cultured in RPMI media supplemented with 10% FBS (Sigma-Millipore). MCF10A cells were cultured in DMEM/F-12 media containing 5% horse serum, 20 ng/mL EGF, 10 μg/mL insulin, 0.5 μg/mL hydrocortisone, and 100 ng/mL cholera toxin. MDA-MB-231 cells were a gift from P. Siegel [McGill University, Montreal, Canada (16)] and were cultured in DMEM with 10% FBS. GFP-tagged ezrin (pEGFP-N1) was a gift from M. Arpin (Curie Institute, Paris). GFP-tagged moesin (pEGFP-N1) was obtained from Addgene (pHJ320 plasmid: 20671). Radixin cDNA was isolated from MDA-MB-231 cells and was cloned into the pEGFP-N1 vector. Dicer siRNA (dsiRNA) targeting ezrin, radixin, or moesin, as well as a nontargeting control dsiRNA, were purchased from Integrated DNA Technologies. For xenograft studies, a GFP-expressing MDA-MB-231 cell line, transduced with a pGIPZ nontargeting lenti-viral vector (Open Biosystems), was used for biophotonics imaging of lung metastasis. All cell lines were cultured free of antibiotics/antimycotics and were determined to be *Mycoplasma* negative by monthly PCR testing using the following primers: Forward: 5′-GGGAGCAAACAGGATTAGATACCCT-3′, Reverse: 5′-TGCACCATGTGTCACTCTGTTAACCTC-3″. Cell line authentication was not performed for this study. After initial thawing, cells were maintained in culture for no more than 2 months or less than 15 passages.

### The Cancer Genome Atlas and Tissue Microarray Analysis

Ezrin, radixin, and moesin RNA sequencing (RNA-seq) data were obtained from The Cancer Genome Atlas (TCGA) PanCancer Atlas Invasive Breast Carcinoma dataset [*n* = 1,083 (17)] through cBioportal (18, 19). Median z-score values (relative to normal tissue samples) were used to compare *EZR*, *RDX*, and *MSN* expression, as well as *EZR* expression across different molecular breast cancer subtypes. A total of 1,082 samples were available for the analysis. A tissue microarray (TMA) was constructed from a locally accrued, all comer cohort of patients with breast cancer (SEOBC; *n* = 347) as described previously (7). The study was conducted in accordance with Canada's Tri-Council Policy Statement for Ethical Conduct for Research Involving Humans and the Declaration of Helsinki: Ethical Principles for Medical Research Involving Human Subjects. All investigations were performed with prior approval from the Queen's University Health Sciences Research Ethics Board (HSREB) and with written informed consent from all patients. The HSREB is qualified through the Clinical Trials Ontario REB Program and is registered with the U.S. Department of Health and Human Services Office for Human Research Protection. IHC staining was previously performed for estrogen receptor (ER), progesterone receptor (PR), HER2, Ki67, cytokeratin (CK) 5/6, and EGFR and scored by a pathologist as described in ref. 7. On the basis of these results, breast cancer subtypes were categorized as Luminal A (ER and/or PR^+^, HER2*^−^*), Luminal B (ER and/or PR^+^, HER2^+^, or HER2*^−^* plus high Ki-67), HER2^+^ (ER*^−^*/PR*^−^*/HER2^+^), and triple-negative (TN) or basal-like (ER*^−^*/PR*^−^*/HER2*^−^*, or ER*^−^*/PR*^−^*/HER2*^−^* plus positive staining for CK5/6 or EGFR). For the current study, IHC for ezrin was performed on TMA sections as described below. Tumor regions from each core were annotated and automated scoring performed with Halo (Indica Labs, Inc.) using a membrane/cytoplasmic-specific mask. Of the 347 available cases, 269 with sufficient tumor regions were available for analysis. Histo-scores (H-score) were calculated by multiplying the percent positive cells by the staining intensity (low = 1, moderate = 2, high = 3) to yield a H-score range from 0 to 300. These values represent ezrin protein levels in tumor, and were not normalized to ezrin expression in normal/non-neoplastic tissue.

### IHC and Automated Scoring Analysis

IHC was performed on 5 μm–thick formalin-fixed paraffin embedded (FFPE) TMA or mouse tissue sections with cleaved-caspase 3 (Cell Signaling Technology, catalog no. 9661), phospho-Ezrin-Radixin-Moesin (Cell Signaling Technology, catalog no. 3726), or total ezrin (Millipore-Sigma, clone 3C12 catalog no. E8897) using the automated Ventana Discovery XT platform (Ventana Medical Systems) and Ethylenediaminetetraacetic Acid (EDTA) buffer for antigen retrieval (pH 8.0, 100°C). IHC-stained slides were scanned using ScanScope (Aperio Technologies) to obtain digital images. For mouse tissue sections, individual lung metastases were manually annotated and the percent positive cells within metastatic lesions for each marker was assessed by automated scoring with Halo (Indica Labs, Inc.) using a membrane/cytoplasmic-specific algorithm, for cleaved-caspase 3 or phosphorylation site in ezrin, radixin, and moesin (pTERM). Manual annotations also provided total number and mean size (in μm^2^) of metastatic lesions per tissue section. For TMA analysis, total ezrin was scored as described above.

### Immunoblotting

Whole-cell lysates were prepared for SDS-PAGE as described previously (11). Briefly, 10–20 μg of protein were separated by SDS-PAGE, transferred to 0.45 μm polyvinylidene difluoride membranes (Bio-Rad), blocked with 5% nonfat dry milk in 1× TBS/0.1% Tween-20, and then probed with ezrin (Cell Signaling Technology, catalog no. 3145), radixin (Cell Signaling Technology, catalog no. C4G7), moesin (Cell Signaling Technology, clone Q480 catalog no. 3150), phospho-ezrin-radixin-moesin (Cell Signaling Technology, catalog no. 3726), phospho-Serine473 Akt (Cell Signaling Technology, catalog no. 9271), total Akt (Cell Signaling Technology, catalog no. 9272), phospho-Serine136 Bad (D25H8, Cell Signaling Technology, catalog no. 4366), total Bad (Cell Signaling Technology, catalog no. 9292), phospho-Serine536 p65 NFκB (Cell Signaling Technology, catalog no. 3031), total p65 NFκB (Cell Signaling Technology, catalog no. 8242), survivin (Cell Signaling Technology) or γ-tubulin (Sigma-Millipore, catalog no. T5326), with the appropriate secondary antibodies (Cell Signaling Technology).

### Cell Viability and Cleaved-caspase 3 Live Cell Assays

*In vitro* cell viability assays were used to assess cytotoxicity in response to DOX, DTX (SelleckChem), and NSC668394 ezrin inhibitor (NSC, Millipore-Sigma). Briefly, 1.5 × 10^4^ cells were seeded in 96-black well plates (Grenier Bio-One) and were treated for 72 hours with DOX, DTX, or NSC. For combination treatments, DOX or DTX and NSC were added at either a 1:1 molar ratio, or with a fixed concentration of NSC and varying concentrations of DOX or DTX. Cell viability was measured fluorometrically with the PrestoBlue viability reagent (Thermo Fisher Scientific) using a SpectraMax M2 plate reader at 560 nm (excitation) and 590 nm (emission). Half maximal inhibitory concentrations (IC_50_) were generated from nonlinear regression (curve-fit) analysis using GraphPad Prism v9 software. For live detection of apoptotic cells, MDA-MB-231 or ZR-75-1 cells were seeded into 96-well plates and then treated with DOX, DTX, or NSC, alone or in combinations, as described above. To detect apoptotic cells, a caspase 3/7 reagent (CellEvent, Thermo Fisher Scientific, C10423) was added at the time of drug treatment (time 0 hours) which gets cleaved when caspase 3/7 are activated, emitting a fluorescent green nuclear signal. Images were acquired using the IncuCyte live cell imaging system (Essen Biosciences) every 2 hours for a total of 72 hours. The percentage of apoptotic cells was calculated on the basis of the number of fluorescent cells divided by the total number of cells detected by phase contrast.

### Animal Studies

All animal procedures were carried out in accordance with Canadian Council on Animal Care guidelines and with approval of the Queen's University Animal Care Committee. Animals were bred in-house according to the approved protocol (protocol number 2021–2151) and 8–12 weeks old females were used in engraftment studies with drug doses normalized to weights. Breeding pairs of *Rag2*^−/−^*IL2rg*^−/−^ double knockout mice (alymphoid) on a BALB/c background were kindly provided by Dr. M. Ito (Central Institute for Experimental Animals, Kawasaki, Japan).

#### Experimental Metastasis Model

A total of 1 × 10^5^ GFP/MDA-MB-231 cells were intravenously injected into immune-compromised 8–12 weeks old female *Rag2^−/−^IL2rg^−/−^* mice via the lateral tail vein. Treatment with vehicle control (DMSO), DOX (5 mg/kg), DTX (10 mg/kg), or NSC668394 ezrin inhibitor (2 mg/kg) began 5 days postinjection and continued every 7 days (DOX/DTX, total of three doses) or daily (NSC) until approximately day 21. At the study endpoint, lung tissue was harvested and imaged using biophotonics or FFPE for IHC staining. The size and number of lung metastases were analyzed from IHC-stained digital images. Metastatic lesions (defined as >3 tumor cells) were manually annotated for the entire lung section to obtain data on mean metastatic lesion size and relative number of metastases.

#### Spontaneous Metastasis Model

A total of 1 × 10^6^ GFP/MDA-MB-231 cells were injected into the fourth mammary fat pad of 8–12 weeks old female *Rag2^−/−^IL2rg^−/−^* mice in a 1:1 ratio of Matrigel:PBS, using a Hamilton syringe. For neoadjuvant studies, treatment began when tumors reached a palpable size (∼80 mm^3^, ∼day 15 postinjection) and continued for 1 week until tumors were excised (∼day 22 postinjection). Daily caliper measurements of tumor length (*L*) and width (*W*) were taken to calculate tumor volume using the following formula: (π×*L*×*W*^2^)/6. Lung tissue was harvested at approximately day 28 for biophotonics imaging. For neoadjuvant plus adjuvant studies, treatment began at approximately day 15 and continued for approximately 5 days, when primary tumors were resected by recovery surgery. Treatment continued the day after tumor resection until the study endpoint (∼day 28), at which time biophotonics imaging of lung tissue was performed at necropsy.

#### Circulating Tumor Cell Analysis

Peripheral blood was harvested from mice at approximately day 22 following a neoadjuvant treatment schedule as detailed above. Briefly, mice were anesthetized with ketamine (200 mg/kg) and xylazine (10 mg/kg) and blood was harvested using an EDTA-coated syringe with a 27-gauge 5/8″ needle via cardiac puncture. Erythrocytes were lysed using ACK lysis buffer (150 mmol/L NH_4_Cl, 10 mmol/L KHCO_3_, 0.1 mmol/L Na_2_EDTA, pH 7.4). Samples were centrifuged to remove lysed cells, and cell pellets (containing tumor cells, monocytes, etc.) were washed 2× in cold PBS and then fixed in 4% paraformaldehyde. Flow cytometry was performed to detect GFP-positive circulating tumor cells (CTC), using blood from non–tumor-bearing mice as a negative control, and cultured GFP/MDA-MB-231 cells as a positive control (for cell size and fluorescence intensity). Samples were analyzed on a FACS Aria III Cell Sorter (Becton Dickinson), excited at 488 nm and detected at 525 ± 20 nm following forward and side scatter gating to exclude monocytes and cell debris. Results were expressed as the number of CTCs/mL of blood, normalized to the mean of control samples to control for variations between experiments.

### Statistical Analysis

Statistical analyses were performed using GraphPad Prism v.9 software and specifics of each analysis are indicated in the figure and table legends. A *P* < 0.05 was considered statistically significant for all studies.

At the beginning of each animal study, and prior to the engraftment of cancer cells or any drug treatments, animals were assigned to different groups so that each group was comparable based on age and weight. Treatments were administered in a non-blinded manner as some drugs were colored compounds (NSC668394). A total of 52–58 animals per study were used (8–10 per group, 6 groups total per study). For smaller animal studies (testing metastatic burden after neoadjuvant treatment) a total of 16 animals were used (4 per group). Animals were excluded from a study if tumors were considerably smaller or larger than ∼80 mm^3^ at the onset of drug treatment (including vehicle) or if the health of animals during a study required euthanasia as per the approved animal care protocol guidelines.

### Data Availability

Some of the data analyzed in this study came from publicly available datasets: Ezrin, radixin, and moesin RNA-seq data were obtained from TCGA PanCancer Atlas Invasive Breast Carcinoma dataset through at https://www.cbioportal.org/study/summary?id = brca_tcga_pan_can_atlas_2018.

The Supplementary Table of common mutations for the cell lines used was compiled using the Broad Institute Cancer Dependency Map (depmap) online portal (https://depmap.org/portal/depmap/) and SIB Swiss Institute of Bioinformatics Cellosaurus online database (https://web.expasy.org/cellosaurus/).

The SEOBC TMA biomarker data generated are not publicly available due to patient privacy requirements but are available upon reasonable request from the corresponding author. Other data generated in this study are available upon request from the corresponding author.

## Results

### Altering Ezrin Expression Changes the Sensitivity of Breast Cancer Cells to DOX and DTX Treatment *In Vitro*

**FIGURE 1:**
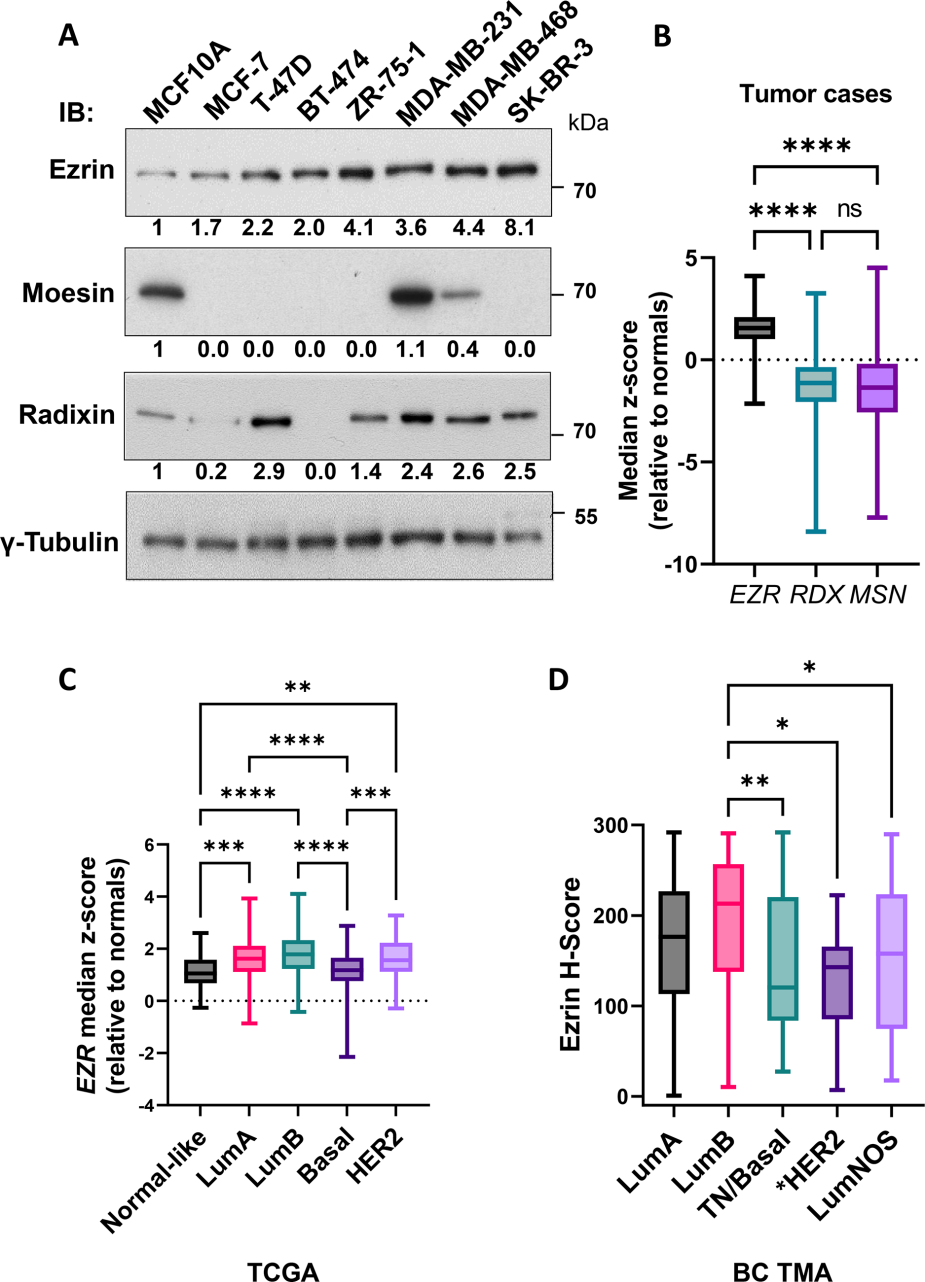
Ezrin expression in breast cancer cell lines and primary tumor samples. **A,** Whole-cell lysates from the indicated breast cancer cell lines and nontumorigenic MCF10A cells were analyzed for total ezrin, radixin, and moesin protein levels by immunoblotting. Tubulin was used as a loading control. Densitometric analysis was performed using ImageJ software and the fold change in ERM protein expression, relative to MCF10A cells, is shown underneath the respective immunoblot. Data are representative of three independent experiments. **B,** Median z-scores for *EZR*, *RDX,* and *MSN* were obtained from TCGA PanCancer Atlas Invasive Breast Carcinoma dataset (*n* = 1,082) as described in Materials and Methods. **C,** Median z-scores for *EZR* across different molecular breast cancer subtypes from TCGA dataset is shown. **D,** Ezrin H-scores were generated from IHC analysis of a breast cancer TMA and were used to assess ezrin protein levels across different breast cancer subtypes. LumA (*n* = 103), LumB (*n* = 70), TN/basal (*n* = 43), LumNOS (Luminal Not-Otherwise-Specified, *n* = 40). The asterisk (*) next to HER2 indicates only 13 cases were available for analysis. *P* values were calculated using one-way ANOVA with Kruskal–Wallis post test. *, *P* < 0.05; **, *P* < 0.01; ***, *P* < 0.001; ****, *P* < 0.0001.

To explore the relationship between ezrin expression and cancer cell sensitivity to chemotherapy agents, we began by assessing ezrin levels in a panel of breast cancer cell lines by immunoblotting. A list of common mutations or gene amplifications related to ezrin signaling for these cell lines can be found in Supplementary Table S1. Ezrin was present in all breast cancer cell lines tested, with the highest relative levels in TN/basal-like MDA-MB-231 and MDA-MB-468 cells, and HER2^+^ SK-BR-3 cells. The nontumorigenic MCF10A breast epithelial cell line expressed lower ezrin levels than all tested breast cancer cell lines (Fig. 1A). No discernable genetic pattern was apparent to explain the variation in ezrin protein levels based on *PI3KCA*, *PTEN, TP53* mutation status or other common mutations in the MAPK pathway or HER2 amplification status (Supplementary Table S1). We also looked at levels of the other ERM family members, radixin, and moesin. Radixin was expressed in most of the breast cancer cell lines, while moesin was only detected in MDA-MB-231 and MDA-MB-468 breast cancer cells, as well as MCF10A cells (Fig. 1A). To extend these observations in clinical samples, we assessed ezrin mRNA and protein expression in primary breast cancer cases using TCGA breast cancer dataset (*n* = 1,082) and a locally accrued TMA cohort (SEOBC, *n* = 347), respectively. We previously reported higher ezrin mRNA and protein in tumor compared with benign tissue (7), consistent with the cell lines data shown here. In our current analyses, we found *EZR* to be the most abundantly expressed ERM family member across all breast cancer cases in TCGA cohort (Fig. 1B). Furthermore, while ezrin is expressed in all breast cancer subtypes, expression was highest in ER^+^ (Luminal A, Luminal B) and HER2^+^ subtypes at the RNA level, and the highest in Luminal A and B subtypes at the protein level (Fig. 1C and D). Ezrin was lowest in TN/basal or normal-like breast cancer cases within TCGA dataset, consistent with the TMA analysis. Because of a low number of HER2^+^ cases within the SEOBC cohort (*n* = 13), we could not reliably correlate ezrin with this breast cancer subtype.

**FIGURE 2:**
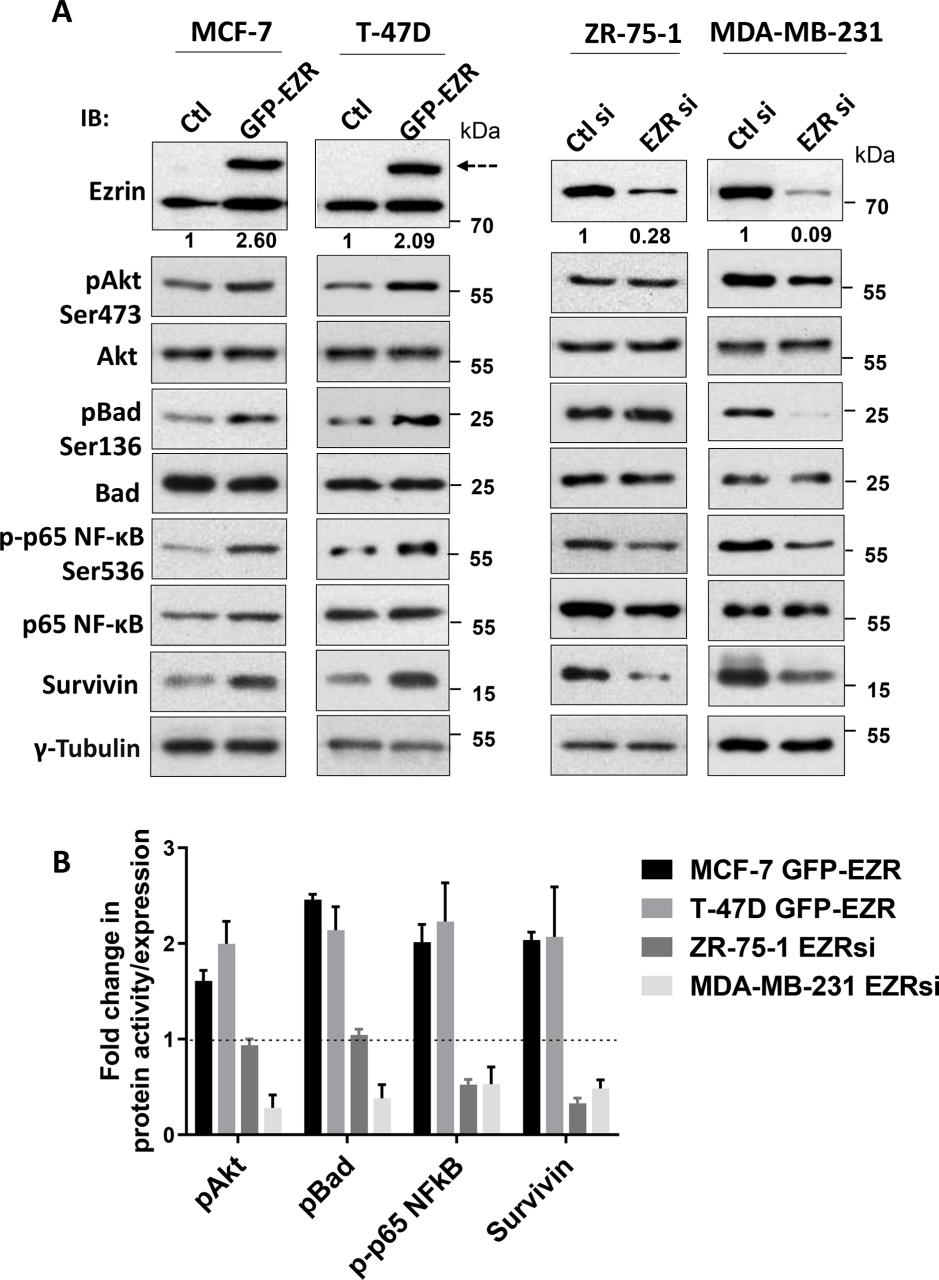
Effects of altered ezrin expression on survival signaling pathways. **A,** MCF-7 and T-47D cells were transfected with either control GFP (Ctl) or GFP-ezrin fusion (EZR) constructs. ZR-75-1 and MDA-MB-231 cells were transfected with either 2 nmol/L scramble control dsiRNA (Ctl si) or ezrin dsiRNA (EZR si). Quantitation of ezrin overexpression or knockdown by densitometry is indicated below the respective immunoblots. At 48 or 72 hours posttransfection (MCF-7/T-47D or ZR-75-1/MDA-MB-231 cells, respectively) cell lysates were prepared and subjected to SDS-PAGE and immunoblotting analysis for the indicated proteins. Dotted arrow indicates exogenous GFP-tagged ezrin levels. **B,** Densitometric analysis was performed to assess changes in protein activity. Phospho-protein levels were normalized to their respective total protein (survivin was normalized to tubulin). Data shown are representative of three independent experiments.

Next, we looked at the cytotoxic sensitivity of these breast cancer cell lines to two commonly used chemotherapy drugs, DOX and DTX. Half-maximal inhibitory concentrations (IC_50_ values) were determined using dose–response cell viability assays in cells treated with chemotherapeutics for up to 72 hours (Supplementary Fig. S1A). While there was no significant correlation between endogenous ezrin levels and chemotherapeutic sensitivity across this panel of breast cancer cells (Supplementary Fig. S1B), experimental manipulation of ezrin expression in selected cell lines was associated with significant alterations in response to chemotherapy drug treatment. Ectopic overexpression of ezrin in MCF-7 and T-47D cells (see Fig. 2A for representative ezrin immunoblots), which exhibited lower endogenous ezrin levels and were highly sensitive to DOX and DTX compared with other breast cancer cells, was associated with 5- to 10-fold increases in IC_50_ values, indicating increased resistance to DOX or DTX, compared with control cells (empty vector; Table 1). In contrast, ectopic over expression of either radixin or moesin in these same cells (Supplementary Fig. S2) did not significantly affect their sensitivity to DOX or DTX.

**TABLE 1:** Manipulation of ezrin changes the sensitivity of breast cancer cells to DOX and DTX

	Control	WT EZR	WT RDX	WT MSN
MCF-7^a^				
DOX (μmol/L)	2.50 ± 1.02	14.28 ± 7.20***	3.49 ± 1.06	2.64 ± 1.53
DTX (nmol/L)	2.43 ± 0.75	37.87 ± 6.19***	0.82 ± 0.55	8.08 ± 2.46
T-47D^a^				
DOX	1.77 ± 0.59	13.88 ± 5.88*	0.60 ± 0.43	1.63 ± 0.10
DTX	1.30 ± 0.34	10.39 ± 4.51***	0.88 ± 0.60	4.59 ± 2.47
	**Controlsi**	**EZRsi**	**RDXsi**	**MSNsi**
ZR-75-1^b^				
DOX (μmol/L)	17.45 ± 2.76	4.84 ± 2.71***	25.66 ± 1.46*	—
DTX (nmol/L)	51.57 ± 4.41	9.70 ± 3.02***	63.71 ± 3.42	—
MDA-MB-231^b^				
DOX	1.48 ± 0.30	0.45 ± 0.14***	2.53 ± 0.51**	1.06 ± 0.20
DTX	99.77 ± 12.02	10.56 ± 2.78***	75.73 ± 10.01	43.78 ± 9.21***

^a^MCF-7 or T-47D cells were transfected with either GFP-fused ezrin (EZR), radixin (RDX), moesin (MSN), or empty vector (control) constructs and were then treated with DOX or DTX for 72 hours. See Fig. 2A and Supplementary Fig. S2 for immunoblotting analysis of exogenous protein levels. Cell viability analysis was performed, and nonlinear (curve-fit) analysis was conducted to generate IC_50_ values using GraphPad Prism as described in Materials and Methods.

^b^ZR-75–1 or MDA-MB-231 cells were transfected with 2 nmol/L siRNA against ezrin (EZRsi), radixin (RDXsi), moesin (MSNsi) or nontargeting control siRNA (Controlsi) and were then treated with DOX or DTX for 72 hours. See Fig. 2A and Supplementary Fig. S2B and C for quantitation of knockdown levels. Cell viability and curve-fit analysis was performed as described previously. ZR-75–1 cells do not express MSN and therefore no IC_50_ values are reported for MSNsi knockdown in this cell line. Data shown are representative of at least three independent experiments (MCF-7/T-47D data: *n* = 3 for control; *n* = 3/4 for ezrin overexpression; *n* = 3/4 for moesin overexpression; *n* = 3 for radixin overexpression. ZR-75–1/MDA-MB-231 data: *n* = 3 for all groups). *, *P* < 0.05; **, *P* < 0.01; ***, *P* < 0.001, when compared with control using two-way ANOVA with Tukey post test. Bolded values represent those comparisons that were statistically significant compared to control.

Next, we depleted ezrin using siRNA in MDA-MB-231 and ZR-75-1 breast cancer cells, which have high ezrin levels and exhibit greater resistance to DOX and DTX compared with MCF-7 or T-47D cells and assessed the effect on chemotherapeutic drug sensitivity (see Fig. 2A for ezrin representative immunoblots and quantitation of knockdown levels). Depleting ezrin markedly increased the sensitivity of MDA-MB-231 and ZR-75-1 cells to DOX and DTX treatment, as demonstrated by 3- to 10-fold reduction in IC_50_ values compared with controls (Table 1). We also tested the effects of depleting radixin and moesin on the sensitivity of these cells to DOX and DTX treatment (Table 1; Supplementary Fig. S2). Intriguingly, radixin depletion slightly reduced sensitivity to DOX in both ZR-75-1 or MDA-MB-231 cells, as indicated by the approximately 1.5-fold higher IC_50_ values, but it did not significantly affect sensitivity to DTX (Table 1). In contrast, moesin depletion in MDA-MB-231 cells did not significantly affect their sensitivity to DOX treatment; however, it did increase their sensitivity to DTX compared with control cells, although to a lesser extent than when ezrin was depleted (∼50% vs. ∼90%, respectively; Table 1). Collectively, these observations suggest that ezrin is the predominant ERM protein involved in modulating chemosensitivity of breast cancer cells to these cytotoxic drugs.

### Altering Ezrin Expression Changes the Activity of Key Survival Signaling Pathways

Because ezrin is linked to the PI3K/Akt and NFκB signaling pathways, which play roles in promoting survival and metastasis (20–22), we next asked whether altering ezrin expression would be associated with changes in the activity of these pathways when exposed to a chemotherapeutic drug challenge. We transiently overexpressed GFP-tagged ezrin in low-ezrin expressing MCF-7 and T-47D cells, and depleted ezrin in high-ezrin expressing MDA-MB-231 and ZR-75-1 cells. We then assessed activation markers of PI3K and NFκB signaling in the presence of DOX by immunoblotting analysis (0.5 μmol/L; Fig. 2). Phospho-Akt, phospho-Bad, and phospho-p65 NFκB were elevated by approximately 2-fold in ezrin overexpressing MCF-7 and T-47D cells; whereas phospho-p65 NFκB levels were reduced by approximately 50% in ezrin-depleted ZR-75-1 and MDA-MB-231 cells (Fig. 2). Interestingly, neither Akt nor Bad activity was affected by ezrin depletion in ZR-75-1 cells. However, phospho-p65 NFκB, phospho-Akt and phospho-Bad were all reduced by approximately 50% in ezrin-depleted MDA-MB-231 cells (Fig. 2). Survivin, part of the inhibitor of apoptosis (IAP) family of anti-apoptotic proteins and downstream of both PI3K and NFκB pathways, was increased in ezrin-overexpressing MCF-7 and T-47D cells and reduced in ezrin-depleted ZR-75-1 and MDA-MB-231 cells (Fig. 2). Together, these data support the role of ezrin in regulating key survival pathways.

### Anti-ezrin Treatment Sensitizes Breast Cancer Cells to DOX and DTX Treatment *In Vitro*

**FIGURE 3:**
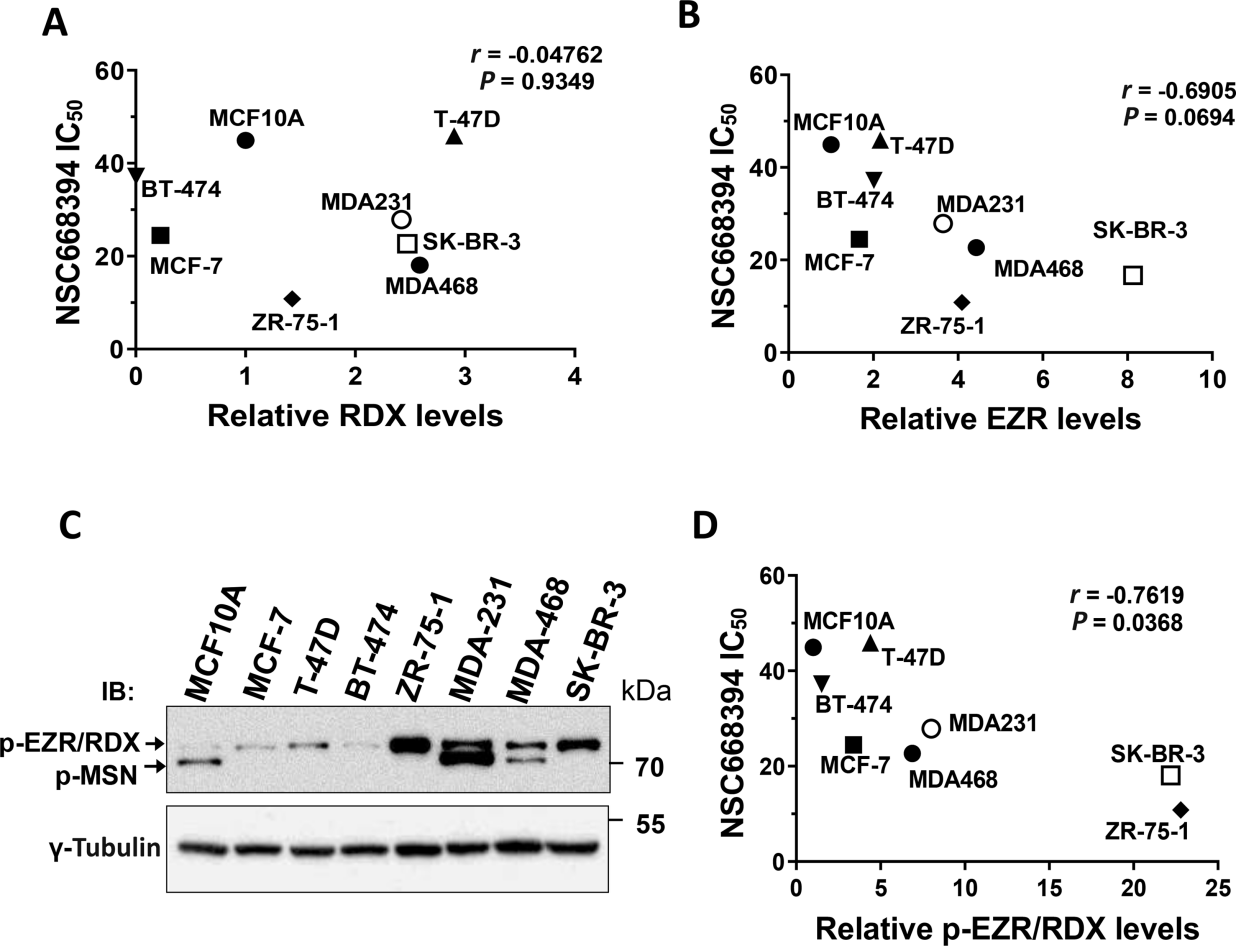
Association of ERMs with NSC sensitivity. **A** and **B,** Relative RDX or EZR levels calculated from Fig. 1A were graphed against the indicated cell line NSC IC_50_ values. Spearman correlation analysis was performed using Graphpad Prism and the respective *r* and *P* values for each analysis are indicated. **C,** Immunoblotting analysis was performed for phospho-ERMs for the panel of cell lines shown. **D,** Relative p-EZR/RDX levels were calculated from **D** and graphed against the indicated cell line NSC IC_50_ values from Supplementary Table S2. Because of the similar molecular weights of p-EZR and p-RDX, γ-tubulin was used to normalize p-EZR/p-RDX values which were then made relative to MCF10A as a control. Spearman correlation analysis was performed as described above. Data shown are representative of three independent experiments.

Bulut and colleagues previously demonstrated the efficacy of blocking ezrin function using small-molecule inhibitors in preclinical osteosarcoma models of metastasis (8). We recently utilized one of these inhibitors, NSC668394 (NSC), which preferentially binds to and inhibits ezrin activity over other ERMs (8) and showed that it reduced ezrin activity *in vitro* and attenuated cancer cell migration and lymph node metastasis *in vivo* (7). We therefore wanted to assess whether treating breast cancer cells with NSC would alter their sensitivity to DOX or DTX treatment. We first explored the cytotoxicity of NSC alone and determined the IC_50_ values for each breast cancer cell line (Supplementary Table S2). We then looked for correlations between NSC sensitivity and expression levels of ezrin and radixin in this panel of breast cancer cells. Radixin levels showed no correlation with NSC sensitivity (Spearman rho = −0.04762; *P* = 0.9349; Fig. 3A); however, ezrin levels trended closely toward a significant negative correlation (Spearman rho = −0.6905; *P* = 0.0694; Fig. 3B). Moesin was excluded from this analysis because only MDA-MB-231 and MDA-MB-468 expressed detectable levels.

Because the active form of these three ERM proteins is phosphorylated at the conserved regulatory threonine residue in the C-terminal domain, we performed immunoblotting using an antibody that recognizes this pTERM and repeated the correlation analysis with NSC sensitivity (Fig. 3C and D). When restricted to the band at the position of comigrating phospho-ezrin (p-EZR) and phospho-radixin (p-RDX), a significant negative correlation was observed between NSC sensitivity and the pEZR/RDX signal (Spearman rho = −0.7619; *P* = 0.0368; Fig. 3D). This closely matches the values for total ezrin (Fig. 3B), but not total radixin (Fig. 3A), supporting the conclusion that ezrin expression and activation state correlates with NSC sensitivity in this panel of breast cancer cell lines. The analysis of phospho-moesin could not be performed with only two data points.

**FIGURE 4:**
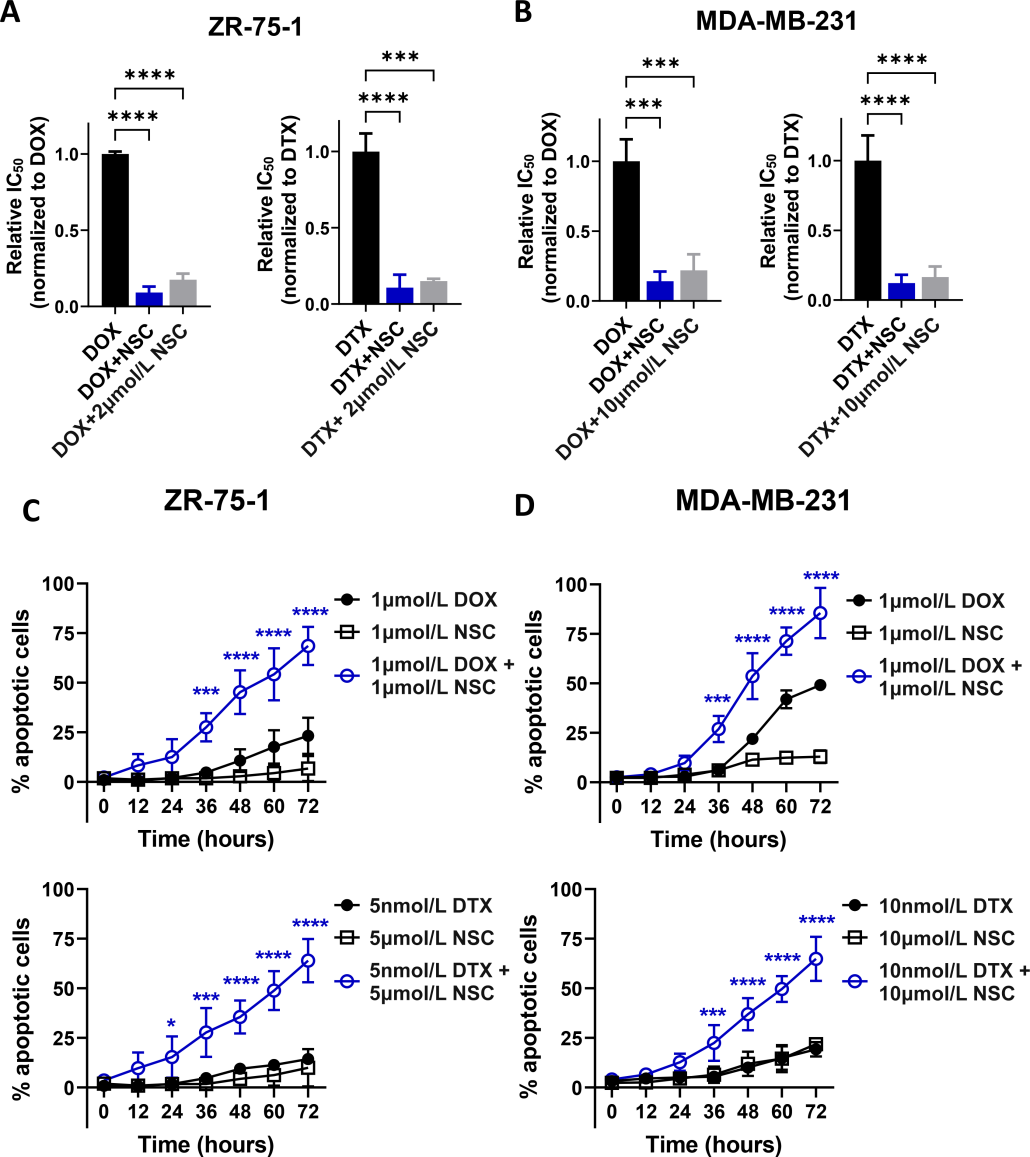
Ezrin inhibition sensitizes breast cancer cells to DOX and DTX. **A** and **B,** ZR-75-1 or MDA-MB-231 cells were treated for 72 hours with varying concentrations of either DOX or DTX alone, or in combination with NSC. NSC was either combined at 1:1 or 1,000:1 molar ratios with DOX or DTX, respectively (DOX+NSC, DTX+NSC), or kept constant at the indicated NSC IC_15_ value of the corresponding cell line (DOX/DTX+NSC 2 μmol/L for ZR-75-1 cells, DOX/DTX+10 μmol/L NSC for MDA-MB-231 cells). Cell viability was measured as described in Materials and Methods. IC_50_ values (relative to DOX or DTX alone), were generated from nonlinear regression (curve-fit) analysis using GraphPad Prism. **C** and **D,** Live cell imaging was performed on ZR-75-1 or MDA-MB-231 cells to detect the levels of caspase-3/7 across the treatment groups and times, as shown. The percentage of apoptotic cells was calculated on the basis of the number of fluorescent nuclei detected in the wells divided by the total number of cells present, by phase contrast. Data are representative of three independent experiments. *P* values were generated using one-way ANOVA with Tukey post test. *, *P* < 0.05; ***, *P* < 0.01; ****, *P* < 0.0001.

We next examined the sensitivity of MDA-MB-231 and ZR-75-1 cells to DOX or DTX in the absence or presence of NSC (at molar ratios of 1:1 for DOX plus NSC, or 1:1,000 for DTX plus NSC). This analysis showed a markedly increased sensitivity of MDA-MB-231 and ZR-75-1 cells to both DOX and DTX treatment, as demonstrated by the 5- to 10-fold reduction in IC_50_ values for these cell lines (Fig. 4A and B). Similarly, treating cells with increasing concentrations of DOX or DTX and a fixed dose of NSC (based on the NSC IC_15_ for each cell line), showed that NSC markedly sensitized MDA-MB-231 and ZR-75-1 cells to DOX and DTX treatment (Fig. 4A and B). We evaluated these drug interactions using two well-known methods, combination index and excess-over-bliss analyses (23, 24). Both methods demonstrated drug synergy between NSC and DOX/DTX treatments over a wide range of drug concentrations, particularly between 1 and 25 μmol/L for DOX and 5–50 nmol/L DTX (Supplementary Table S3). Assessment of apoptosis in drug-treated cells, using a fluorescent caspase 3/7 marker, revealed a 60%–80% increase in apoptotic cells in the combination treatments of NSC with DOX or DTX compared with NSC or chemotherapy drug alone (Fig. 4C and D), indicating that ezrin inhibition enhanced the cytotoxic effects of DOX and DTX.

### Anti-ezrin Treatment Sensitizes Metastatic Breast Cancer Cells to DOX and DTX Treatment *In Vivo*

**FIGURE 5:**
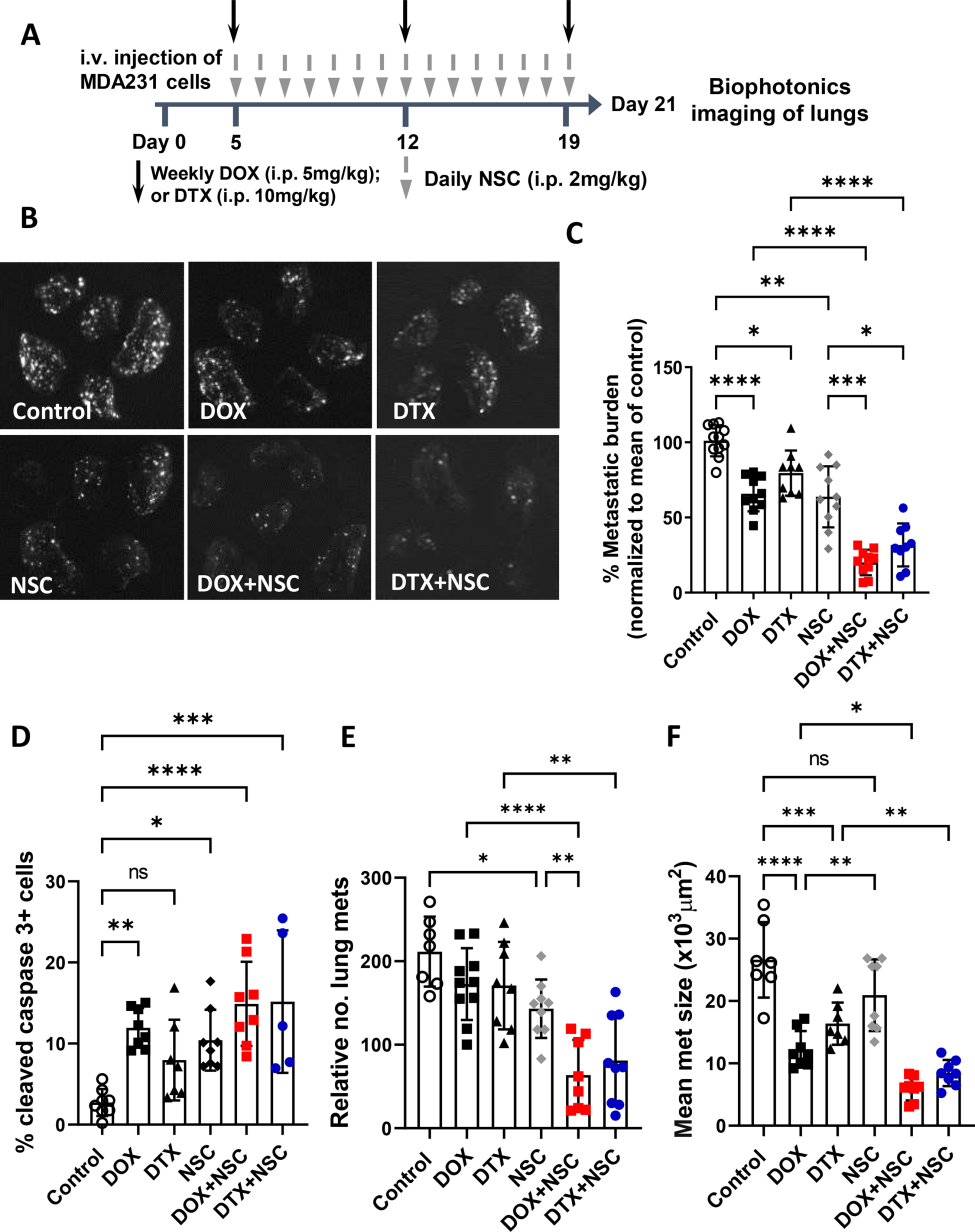
Ezrin inhibition sensitizes metastatic breast cancer cells to DOX and DTX in an experimental metastasis model. **A,** Treatment schedule for the experimental metastasis model. GFP-expressing MDA-MB-231 cells (1 × 10^5^) were injected into the tail veins of immune-compromised mice and were treated starting at day 5 post-injection with DOX or DTX alone (weekly) or in combination with NSC (daily) at the indicated doses. **B,** Biophotonics imaging was performed on harvested lungs at the study endpoint (∼day 21 after intravenous injection). **C,** Metastatic burden, defined as total tumor area divided by total lung area, was assessed and normalized to the mean of the control group in order to allow pooling of data from separate experiments. *N* = 8–10 per group. Lungs were processed and either stained for cleaved caspase-3 by IHC (**D**) or analyzed for the number and size of metastases using digital histology images (**E** and **F**) for each treatment group as described in Materials and Methods. *N* = 7–8 per group for cleaved caspase-3 except for the DTX+NSC treatment, *N* = 5, due to lack of detectable metastatic lesions in some tissue sections. *N* = 8–10 per group for metastasis number and size analyses. *P* values were calculated using one-way ANOVA with Tukey post test. *, *P* < 0.05; **, *P* < 0.01; ***, *P* < 0.001; ****, *P* < 0.0001.

Metastatic cancer cells are often resistant to chemotherapy (25) and we therefore asked whether NSC treatment could sensitize metastatic breast cancer cells to DOX or DTX in an experimental metastasis model which mirrors adjuvant treatment (i.e., in the absence of a primary tumor). We intravenously injected GFP-expressing MDA-MB-231 cells into mice to seed the lungs. Five days later, mice were treated for 15 days as indicated, and at the study endpoint lungs were harvested for quantitation of metastatic burden (Fig. 5A). Using this model, we observed a modest, approximately 40% reduction of metastatic burden with DOX treatment alone; which was similar to single-agent treatment with NSC (Fig. 5B and C). Treatment with DTX alone reduced metastatic burden to a lesser extent, by approximately 20%. When mice were treated with NSC plus DOX or DTX, we observed an improved reduction in metastatic burden, by approximately 80% and 70%, respectively, relative to vehicle control–treated mice (Fig. 5B and C). We also assessed active ezrin levels within lung metastases by IHC and found that NSC treatment reduced pTERM levels in the metastatic lesions, with minimal effects to pTERM levels in the surrounding lung epithelium (Supplementary Fig. S3). This suggests a tumor-cell specific effect of NSC *in vivo*. IHC analysis of cleaved caspase-3 staining in lung metastases revealed significantly higher percentages of apoptotic cells in all the drug treatment groups compared with control-treated mice except for DTX treatment. While the percentage of apoptotic cells was higher for both DOX+NSC (14.9%) and DTX+NSC (15.2%) treatment groups compared with single-agent treatments (11.9%, 8.0%, and 10.4% for DOX, DTX, and NSC, respectively), these differences did not reach statistical significance (Fig. 5D). Histologic assessment of lung metastases revealed differing effects of each treatment on the number and size of metastatic lesions; single-agent DOX and DTX treatment reduced the size but not the total number of metastases present while NSC treatment on the other hand, reduced the total number of metastatic lesions but had minimal effect on their size. Both DOX+NSC and DTX+NSC combination treatments resulted in significant reductions in both the number and size of metastases compared with all monotherapies (Fig. 5E and F). Thus, in this experimental metastasis model, all three single-agent treatments reduced the burden of metastasis to a similar extent. However, the data suggest that they have differential effects on the survival/outgrowth of microscopic metastatic lesions.

### Neoadjuvant Chemotherapy–induced Metastasis is Prevented by Anti-ezrin Treatment

**FIGURE 6:**
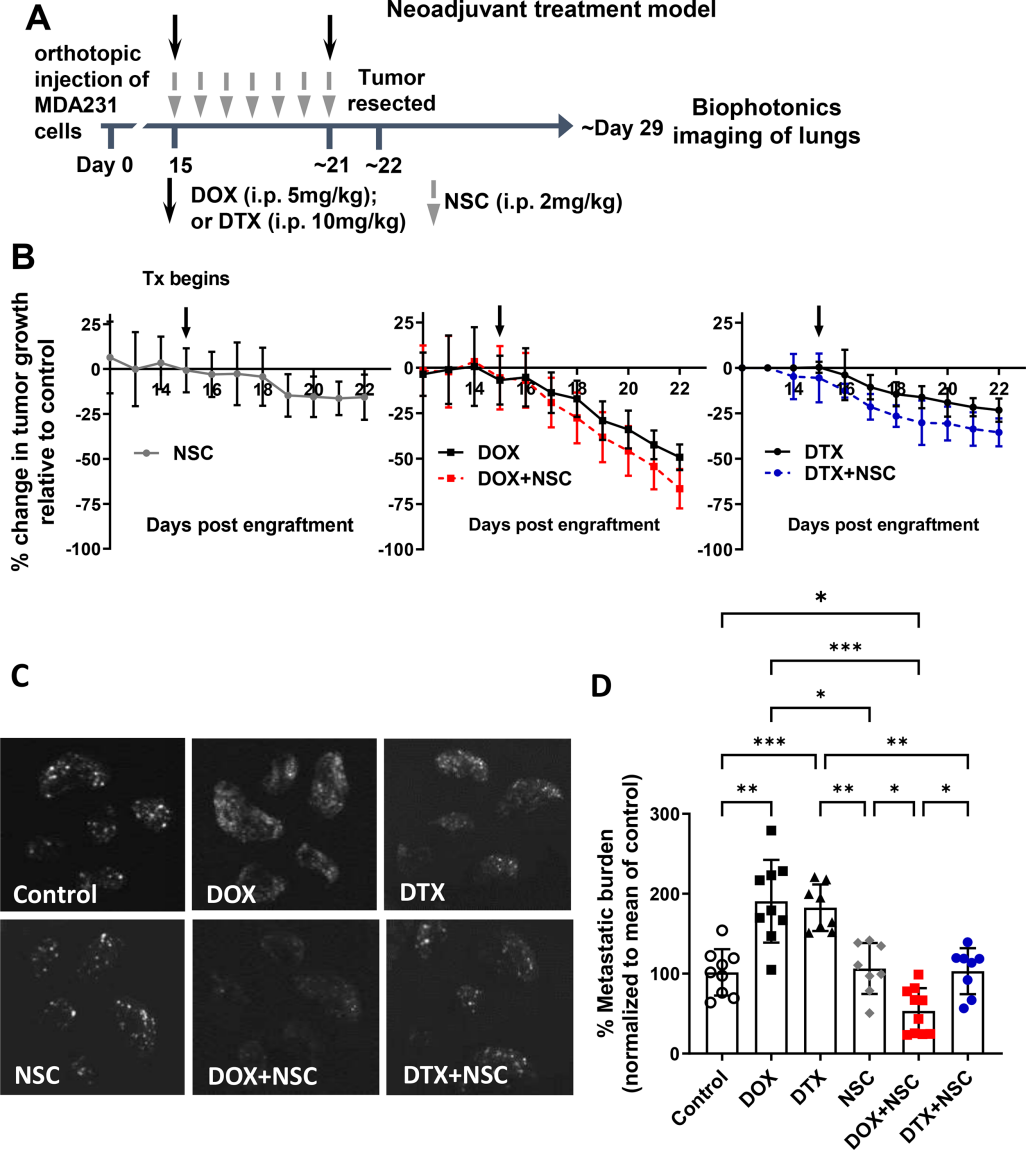
Ezrin inhibition abrogates neoadjuvant chemotherapy-induced metastasis. **A,** Treatment schedule for neoadjuvant model. GFP-expressing MDA-MB-231 cells were orthotopically engrafted (1 × 10^6^) into immune-compromised mice. The indicated treatment began once primary tumors reached approximately 80 mm^3^ in size (∼day 15 post-engraftment) and continued as shown until tumors were resected at approximately day 22. No treatment was administered after resections. **B,** Tumor growth was monitored daily by caliper measurements and changes in tumor volumes for each treatment group were calculated relative to the growth of tumors in the control group. Downward arrows indicate when treatments were administered. **C,** Lungs were harvested at day 30 for biophotonic imaging. **D,** Metastatic burden was calculated and normalized to the mean of the control group. *P* values were calculated using one-way ANOVA with Tukey post test. *, *P* < 0.05; **, *P* < 0.01; ***, *P* < 0.001; ****, *P* < 0.0001. *N* = 8–10 per group.

Despite having an anti-tumor effect, neoadjuvant chemotherapy has been shown to promote cancer cell dissemination in preclinical models (3, 4). Given our observations demonstrating that NSC sensitizes cancer cells to DOX and DTX treatment, we next asked whether NSC could block neoadjuvant DOX- or DTX-induced metastasis. Orthotopically engrafted GFP-expressing MDA-MB-231 cells were allowed to grow primary tumors to a size of approximately 80 mm^3^, at which point mice were given neoadjuvant treatment for 1 week, followed by resection of primary tumors and an additional 7 days with no treatment before the end of the study, as detailed in Fig. 6A. Importantly, treatment began at approximately 15 days post-engraftment, a time when tumor cells had already started to metastasize to the lungs (Supplementary Fig. S4A and S4B). This allowed us to examine the effects of the neoadjuvant treatment on existing microscopic metastasis or the promotion of metastasis. Assessment of primary tumor growth revealed that tumors regressed by approximately 50% in mice treated with DOX and by approximately 23% with DTX treatment compared with vehicle control-treated mice, while NSC treatment reduced tumor size by approximately 15% relative to control (Fig. 6B). The combination of NSC with DOX or DTX did not significantly reduce tumor growth beyond what was observed with DOX or DTX alone (Fig. 6B).

When we assessed the effects of neoadjuvant treatment on metastasis, we observed a marked increase in metastasis with neoadjuvant DOX or DTX treatment alone (Fig. 6C and D); and this was observed despite primary tumors showing signs of regression, particularly with DOX treatment (Fig. 6B). Neoadjuvant NSC treatment alone had no effect on metastasis; more importantly however, the addition of NSC to neoadjuvant DOX or DTX treatment was associated with significantly reduced metastatic burden relative to DOX or DTX monotherapy, with the DTX+NSC treatment reducing metastatic burden to levels comparable with vehicle control–treated mice, and DOX+NSC lowering metastasis by nearly 50%, relative to the vehicle control group (Fig. 6D). Given the timing of when metastasis was assessed in these studies (∼7 days after neoadjuvant treatment and tumor resection), we also looked at metastatic burden at the end of neoadjuvant treatment in a separate study using DOX alone or in combination with NSC (Supplementary Fig. S4C and S4D). While overall detectable metastasis was lower in this study compared with our previous neoadjuvant model across all treatment groups (∼4-fold lower), we consistently observed that neoadjuvant DOX increased metastatic burden, and the addition of NSC to DOX treatment prevented neoadjuvant treatment–induced metastasis.

Previous studies have also shown that neoadjuvant chemotherapy–induced metastasis is associated with increased levels of CTCs (4). Consistent with those studies, we observed that DOX- and DTX-treated tumor-bearing mice had higher CTC levels compared with vehicle control–treated mice, while NSC-treated mice showed CTC levels comparable with control (Supplementary Fig. S5). However, the addition of NSC to DOX or DTX treatment did not significantly reduce CTC levels relative to single treatment DOX and DTX groups. Thus, while neoadjuvant anti-ezrin only treatment did not affect CTCs, it also did not eliminate or prevent DOX- or DTX-induced CTC levels.

### Anti-ezrin Treatment in Combination with DOX or DTX Reduces Overall Metastatic Burden in a Neoadjuvant Plus Adjuvant Treatment Model

**FIGURE 7:**
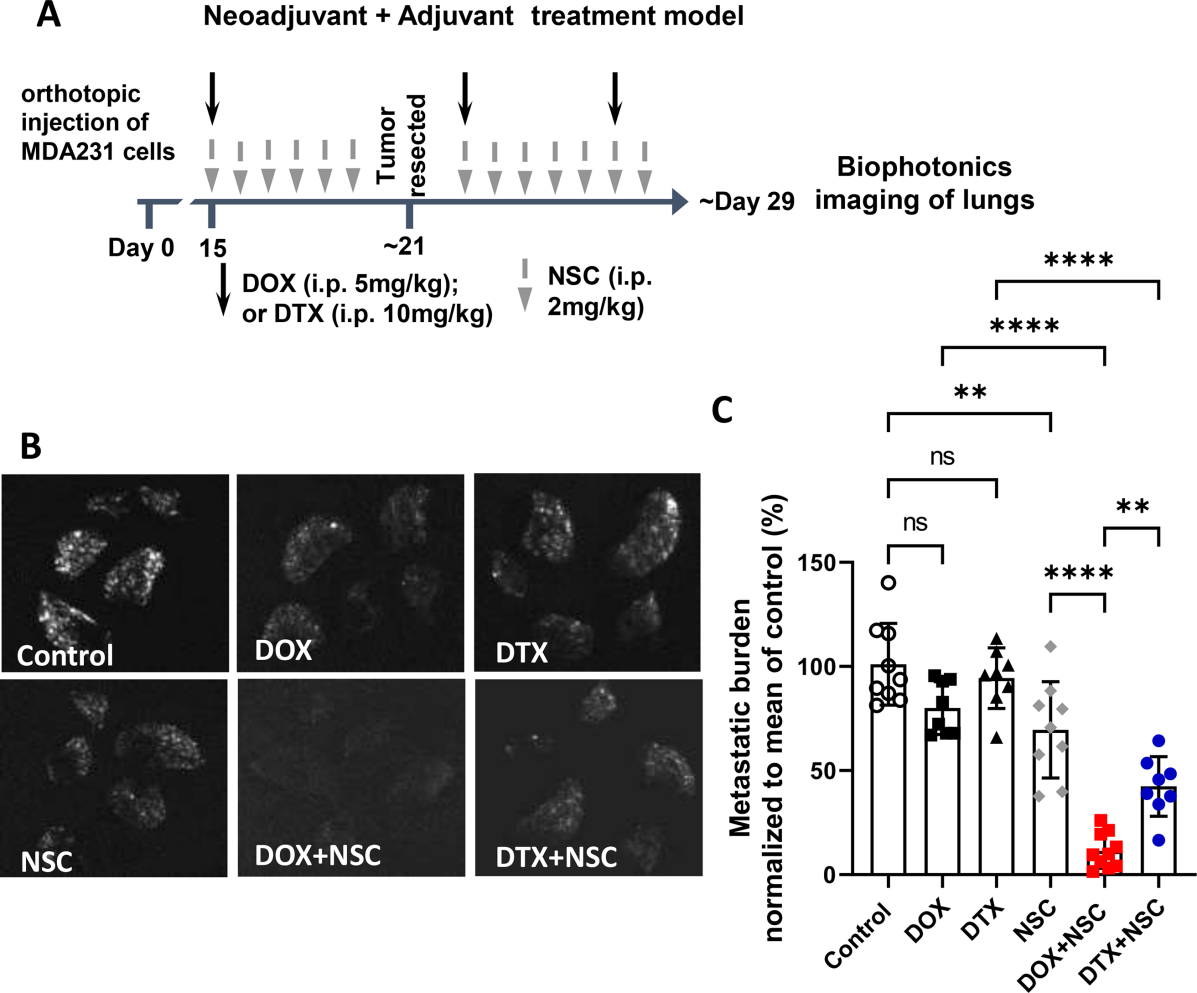
Ezrin inhibition sensitizes metastatic breast cancer cells to DOX and DTX in a neoadjuvant plus adjuvant treatment model. **A,** Treatment schedule for neoadjuvant plus adjuvant model. GFP-expressing MDA-MB-231 cells were orthotopically engrafted into immune-compromised mice (1 × 10^6^). The indicated treatments (downward arrows) began once primary tumors reached approximately 80 mm^3^ in size (∼day 15 post-engraftment) until tumors were resected at day 21 and continued post-resection as shown. **B,** Lungs were harvested at approximately day 29 post-engraftment for biophotonics imaging. **C,** Metastatic burden was calculated and normalized to the mean of the control group. *P* values were obtained using one-way ANOVA with Tukey post test. *, *P* < 0.01; ****, *P* < 0.0001. *N* = 8–10 per group.

To determine whether continuing anti-ezrin treatment after tumor resection had any further effect on reducing metastasis, we next evaluated the efficacy of NSC in a neoadjuvant plus adjuvant treatment model, as detailed in Fig. 7A. In this setting, neoadjuvant plus adjuvant NSC alone reduced metastasis by approximately 25% relative to control, while metastatic burden in DOX- or DTX-treated mice was similar to control mice (Fig. 7B and C). However, when NSC was given in combination with DOX or DTX treatment, metastatic burden was reduced by approximately 90% with DOX and by approximately 60% with DTX treatment, compared with control mice (Fig. 7C); and these effects were significantly greater than DOX or DTX alone. These results revealed that the addition of anti-ezrin treatment to chemotherapy markedly reduced overall metastatic burden, and this benefit of anti-ezrin treatment was apparent in both the neoadjuvant and the neoadjuvant plus adjuvant settings.

Because the number of neoadjuvant DOX/DTX doses differed in this neoadjuvant plus adjuvant model compared with our neoadjuvant only treatment model (one neoadjuvant dose of DOX or DTX vs. two, respectively), we also evaluated metastatic burden at the end of neoadjuvant treatment (∼day 21) in a smaller, separate study using only DOX and NSC (Supplementary Fig. S4D). This revealed that a single neoadjuvant dose of DOX was able to enhance metastasis to a similar extent as our neoadjuvant only model with two doses of DOX (∼2-fold increase relative to control; Supplementary Fig. S4C), and the addition of NSC to DOX treatment prevented neoadjuvant DOX-induced metastasis, thus validating the findings from our neoadjuvant only treatment mode (Fig. 6). Furthermore, it demonstrates that the lower metastatic burden observed across all drug treatment groups in the neoadjuvant plus adjuvant model can be attributed to the adjuvant therapy. Collectively, these results reveal that anti-ezrin treatment markedly reduces the overall metastatic burden in the setting of both neoadjuvant and adjuvant chemo-therapy.

## Discussion

While current cytotoxic therapies are aimed at reducing primary tumor burden, few are effective at preventing or curing metastatic disease, the main cause of death for patients with cancer. Furthermore, emerging evidence indicates that neoadjuvant chemotherapy may actually promote metastasis (3, 4). Here, we provide evidence that ezrin inhibition sensitizes metastatic breast cancer cells to anthracycline and taxane treatment both *in vitro* and *in vivo*. We also demonstrate that systemic anti-ezrin therapy reduces neoadjuvant chemotherapy–induced metastasis; and with the addition of adjuvant treatment, anti-ezrin therapy substantially reduces overall metastasis in combination with DOX or DTX. It is important to note that we designed our *in vivo* treatment models to assess the effects of these treatments on existing microscopic metastases, as opposed to preventing metastatic events from occurring. The latter has been a well-documented phenomenon for ezrin in several preclinical models, where engraftment of ezrin-deficient or inactive ezrin mutant cell lines *in vivo* is associated with reduced metastasis (9, 11, 26); however, little was known about the effect of targeting ezrin on already established micrometastases, as occurs in a “real-life” clinical scenario. Our data indicate that the chemosensitizing effect of anti-ezrin therapy largely affects disseminated cancer cells at their secondary organ site, as no significant changes in primary tumor regression or CTC levels were observed with the addition of NSC to DOX or DTX treatment. These results are consistent with previous findings from our group and others demonstrating a metastatic-specific function of ezrin (9, 11, 12, 26).

Resistance to chemotherapy is a common feature of metastatic cancer cells for several reasons, whether arising from *de novo* resistance or selected for after multiple rounds of chemotherapy, or through other mechanisms such as tumor dormancy, resistance to apoptosis, or increased DNA repair (1, 27). While ezrin is known to regulate survival signaling, its role in drug resistance is not fully understood. Nonetheless, studies have demonstrated the ability of ezrin to promote drug resistance in both solid and blood-based cancers through different mechanisms. For instance, multidrug resistance proteins such a P-glycoprotein (P-gp) require ezrin for their proper localization and drug-efflux functions in cancer cells (28, 29); and ezrin is implicated, along with other ERM members, in the transfer of P-gp–mediated drug resistance to previously drug-sensitive breast cancer cells via extracellular vesicles (30, 31). Other studies showed the ability of ezrin to augment oncogenic EGFR and HER2 signaling in non–small cell lung cancer and breast cancer cells, respectively, while pharmacologic inhibition of ezrin with the same small molecules initially identified by Bulut and colleagues (8), synergistically enhanced erlotinib- and lapatinib-mediated killing of tumor cells (32, 33). These same ezrin inhibitors have recently been shown to reduce cell viability and cell-cycle progression of acute myeloid leukemia (AML) cells (34) and may also demonstrate synergy with chemotherapy and/or targeted therapies used to treat AML.

Limited clinical evidence demonstrating an association between ezrin and chemotherapy resistance has been found. However, one study showed increased ezrin expression in CHOP (cyclophosphamide, DOX, vincristine, prednisone) resistant diffuse large B-cell lymphoma patient samples; and that downregulating ezrin expression (either directly or by overexpressing miR-148b) attenuated CHOP resistance both *in vitro* and *in vivo* (35). We attempted to assess whether short-term (up to 24 hours) exposure to chemotherapy drugs could alter ezrin or active-ERM levels and did not observe any statistically significant changes in either total or active protein (Supplementary Fig. S6). While we could not evaluate whether ezrin levels are upregulated in acquired chemotherapeutic drug-resistant breast cancer cell lines in our study, we showed that altering ezrin levels can change the sensitivity of breast cancer cells to DOX and DTX treatment *in vitro*. We also showed that changes in ezrin levels are associated with alterations in the activation of PI3K/Akt and NFkB pathways, suggesting that ezrin may be promoting chemotherapy resistance, at least in part, through upregulation of survival signaling. In support of that inference, other studies have linked ezrin to the survival of newly disseminated cancer cells at distant organ sites through these same pathways (12, 20). This may help explain why anti-ezrin treatment in our preclinical models specifically sensitizes metastatic, and not primary tumor cells, to chemotherapy drugs.

In addition to pro-survival pathways, there are likely other mechanisms by which ezrin is involved in chemotherapy resistance. Hypoxia, for instance, plays a well-known role in cancer progression as well as treatment resistance (reviewed in ref. 36); and enhanced ezrin activity has been observed within hypoxic regions of colorectal cancers containing tumor-initiating cells (37). Hypoxia can also affect the intracellular accumulation of chemotherapy drugs as well as their localization (i.e., in acidic lysosomes vs. the nucleus) therefore impacting their cytotoxic effects on cancer cells (36, 38, 39). Thus, further investigation is needed to determine whether ezrin is involved in hypoxia-mediated chemotherapy resistance, as well as other mechanisms of drug resistance.

Given the high degree of homology between ERMs, we also tested whether radixin or moesin play a similar role as ezrin in regulating chemotherapeutic drug sensitivity. Our data show that neither altering radixin nor moesin expression affected sensitivity to DOX or DTX to the same degree as ezrin. Our observations point to ezrin as the predominant ERM protein responsible for modulating sensitivity to anthracycline and taxane treatment in breast cancer cells. While some functional redundancy exists among ERM proteins (40, 41), studies have highlighted distinct but complementary roles for ERMs in regulating the same cellular processes, such as ezrin and moesin in cancer cell invasion (14, 39). Given that both ezrin and moesin depletion resulted in increased sensitivity to taxane treatment, though not explored in this study, it is possible that ezrin and moesin affect sensitivity to taxanes in different ways. It is important to note that ERM activation is regulated by phosphorylation of a conserved threonine residue. Thus, while NSC was initially identified to preferentially bind to and inhibit ezrin phosphorylation (8), some evidence suggests that all three ERMs may be affected (40) and we cannot rule out the possibility NSC may inhibit other ERMs in our model. However, regression analyses suggest a stronger correlation between ezrin levels and NSC sensitivity compared with radixin in breast cancer cells (Fig. 3) and that breast cancer cells may be more dependent on ezrin function over other ERMs.

As tumor cells can spread systemically before therapeutic intervention, for cancers such as breast (5), it is imperative for the overall survival of patients that therapies successfully target both primary tumor and disseminated cancer cell populations. Cytotoxic therapies are by nature designed to target highly proliferative cells. However, Matus and colleagues demonstrated that actively proliferating cell populations, such as those that comprise the bulk of the tumor, are distinct from cells with disseminating potential which are nondividing (41). This suggests that different therapeutic strategies are required to target these different cell populations, especially those disseminated cells which can remain in a quiescent, nonproliferative state for many years. This provides another potential explanation for why chemotherapy alone is ineffective at eliminating metastasis. While we focused on targeting microscopic metastasis, it would be important for future studies to assess whether anti-ezrin treatment can target dormant tumor cells as well.

Accumulating evidence also demonstrates that neoadjuvant chemotherapy treatment alters the tumor microenvironment in ways which promote metastasis and chemoresistance, including the induction of cellular stress in nontumor cells (3) and enhancing tumor invasive characteristics, especially in patients with residual disease (4). Currently, neoadjuvant chemotherapy is mainly used to treat locally advanced breast cancers, but its use has been increasing in patients with early stage disease as well (42). However, evidence shows higher locoregional recurrence rates and no survival benefit in neoadjuvant-treated patients compared with those who received adjuvant chemotherapy only (42). In light of these observations, it will be important to consider future neoadjuvant chemotherapy strategies in combination with anti-metastatic agents that can reduce primary tumor burden while also effectively targeting disseminated cancer cells/micrometastases.

In summary, the data we present here demonstrate the potential of anti-ezrin therapy at targeting metastatic breast cancer cells in combination with chemotherapy and provide rationale for testing its efficacy in other cancer types.

## Supplementary Material

Table S1 Common gene mutations or amplifications found in the BC cell line panel

Table S2 Summary of IC50 values

Table S3 Drug synergy analysis of DOX or DTX in combination with NSC

Figure S1 Correlation analysis of ezrin levels with DOX or DTX sensitivity

Figure S2 Quantitation of exogenous Radixin and Moesin levels or knockdown by siRNA

Figure S3 Automated analysis of pTERM staining in metastatic lesions and adjacent normal lung tissue

Figure S4 Assessment of microscopic metastasis and lung metastatic burden

Figure S5 Anti-ezrin treatment does not alter levels of circulating tumor cells *in vivo*

Figure S6 Ezrin protein expression after DOX and DTX drug challenge
