# Supplementary material for: Targeting the Ezrin Adaptor Protein Sensitizes Metastatic Breast Cancer Cells to Chemotherapy and Reduces Neoadjuvant Therapy–induced Metastasis
Source: Cancer Res Commun. 2022 Jun 17;2(6):456–70. doi: 10.1158/2767-9764.CRC-21-0117 (PMC10010290; doi:10.1158/2767-9764.CRC-21-0117)
Supplement: Table S1 — Common gene mutations or amplifications found in the BC cell line panel [file crc-21-0117-s01.pdf]

Supplementary Table 1

Cell lines	*TP53*	*PTEN*	*PIK3CA*	Other
MCF10A	-	-	-	-
MCF-7	-	-	E545K	PIK3AP1
T-47D	L194F	-	H1047R	
BT-474	E285K	-	K11N	HER2amp
ZR-75-1	-	L108R	-	HRAS
MDA-MB-231	R280K	-	-	BRAF<br>KRAS<br>MYCL<br>PIK3R6
MDA-MB-468	R273H	Silent mut<br>c.253+1 G>T	-	
SK-BR-3	R175H	-	-	HER2amp

Supplementary Table 1: Common gene mutations or amplifications found in the BC cell line panel

A list of mutations/gene amplifications for the breast cell lines used in this study was compiled using the Broad Institute Cancer Dependency Map (depmap) online portal and SIB Swiss Institute of Bioinformatics Cellosaurus online database as described in the Data Availability section of Materials and Methods. The amino acid substitutions are shown for TP53, PTEN and PIK3CA. A PTEN silent mutation is found in MDA-MB-468 cells and the nucleic acid substitution is indicated instead. Other commonly mutated genes or gene amplifications (amp) are listed in the “other” column. Dashes indicate wildtype status of the gene or no known mutation.
