# Supplementary material for: Targeting the Ezrin Adaptor Protein Sensitizes Metastatic Breast Cancer Cells to Chemotherapy and Reduces Neoadjuvant Therapy–induced Metastasis
Source: Cancer Res Commun. 2022 Jun 17;2(6):456–70. doi: 10.1158/2767-9764.CRC-21-0117 (PMC10010290; doi:10.1158/2767-9764.CRC-21-0117)
Supplement: Table S2 — Summary of IC50 values [file crc-21-0117-s02.pdf]

Cell line	NSC IC50 ± SD (μM)
**MCF10A**	**44.95 ± 6.25**
**MCF-7**	**20.67 ± 6.34**
**T-47D**	**45.48 ± 1.17**
**BT-474**	**37.24 ± 1.36**
**ZR-75-1**	**9.43 ± 2.64**
**MDA-MB-231**	**29.32 ± 5.27**
**MDA-MB-468**	**22.67 ± 6.53**
**SK-BR-3**	**16.35 ± 1.32**

Supplementary Table 2: Summary of IC_{50} values
A list of IC_{50} values ± standard deviations (SD) for NSC treatment for the panel of cell lines is shown. The indicated cell lines were treated to varying concentrations of NSC for 72 hours and were subjected to cell viability analysis. IC_{50} values were generated from non-linear regression (curve-fit) analysis using GraphPad Prism software. Mean values and SDs were calculated based on a 3 independent cell viability experiments.
