# Supplementary material for: Targeting the Ezrin Adaptor Protein Sensitizes Metastatic Breast Cancer Cells to Chemotherapy and Reduces Neoadjuvant Therapy–induced Metastasis
Source: Cancer Res Commun. 2022 Jun 17;2(6):456–70. doi: 10.1158/2767-9764.CRC-21-0117 (PMC10010290; doi:10.1158/2767-9764.CRC-21-0117)
Supplement: Table S3 — Drug synergy analysis of DOX or DTX in combination with NSC [file crc-21-0117-s03.pdf]

*Supplementary Table 3*

| | ZR-75-1 | | MDA-MB-231 | |
|---|---|---|---|---|
| DOX (uM) +NSC (uM) | Constant ratio (EOB) | Non-constant ratio (CI) | Constant ratio (EOB) | Non-constant ratio (CI) |
| 0.01 | **0.12** | 5.39 | **0.03** | 9.56 |
| 0.1 | **0.12** | **0.65** | **0.16** | 4.66 |
| 1.0 | **0.24** | **0.65** | **0.41** | **0.32** |
| 5.0 | **0.45** | **0.72** | **0.15** | **0.32** |
| 10 | **0.29** | **0.25** | **0.10** | **0.32** |
| 25 | **0.02** | **0.06** | **0.10** | **0.43** |
| 50 | -0.01 | **0.03** | (0.002) | **0.41** |
| 100 | -0.01 | **0.02** | -0.01 | **0.22** |

| | ZR-75-1 | | MDA-MB-231 | |
|---|---|---|---|---|
| DTX (nM) +NSC (uM) | Constant ratio (EOB) | Non-constant ratio (CI) | Constant ratio (EOB) | Non-constant ratio (CI) |
| 0.01 | **0.09** | 6.91 | -0.08 | 11.73 |
| 0.1 | **0.26** | 2.29 | **0.07** | 2.47 |
| 1.0 | **0.25** | 1.71 | **0.12** | 2.52 |
| 5.0 | **0.52** | **0.79** | **0.21** | 1.63 |
| 10 | **0.33** | **0.79** | **0.20** | **0.7** |
| 25 | -0.11 | **0.88** | **0.29** | **0.73** |
| 50 | -0.02 | **0.32** | **0.03** | **0.69** |
| 100 | (-0.002) | **0.24** | -0.02 | **0.21** |

EOB: Excess over Bliss
CI: Combination Index

**Supplementary Table 3. Drug synergy analysis of DOX or DTX in combination with NSC**

Drug synergy analysis was performed using two different methods: Excess-over-Bliss (EOB; [20] ) and Combination Index (CI; [19]). EOB was performed using a constant ratio of drug 1 to drug 2 across multiple concentrations of drugs tested (i.e. 1:1 with DOX:NSC and 1:1000 with DTX:NSC) while CI analysis was performed using a fixed concentration of NSC (i.e. $IC_{15}$ for ZR-75-1 and MDA-MB-231) with varying concentrations of DOX or DTX. Values in bold text represent concentrations of drug where synergy was observed, whereas negative/italicized values represent concentrations where antagonism was observed. Values in parentheses represent observed additivity.
