# Supplementary material for: Targeting the Ezrin Adaptor Protein Sensitizes Metastatic Breast Cancer Cells to Chemotherapy and Reduces Neoadjuvant Therapy–induced Metastasis
Source: Cancer Res Commun. 2022 Jun 17;2(6):456–70. doi: 10.1158/2767-9764.CRC-21-0117 (PMC10010290; doi:10.1158/2767-9764.CRC-21-0117)
Supplement: Figure S1 — Correlation analysis of ezrin levels with DOX or DTX sensitivity [file crc-21-0117-s04.pdf]

Supplementary Figure 1

A

	DOX	DTX
**Cell line**	**IC50 ± SD (µM)**	**IC50 ± SD (nM)**
MCF-7	1.86 ± 0.85	3.08 ± 1.56
T-47D	0.87 ± 0.33	1.09 ± 0.39
BT-474	8.65 ± 1.22	6.97 ± 1.58
ZR-75-1	18.56 ± 5.20	52.48 ± 4.70
MDA-MB-231	2.63 ± 1.71	106.7 ± 10.81
MDA-MB-468	0.46 ± 0.18	18.23 ± 5.50
SK-BR-3	1.07 ± 0.33	0.04 ± 0.01

B

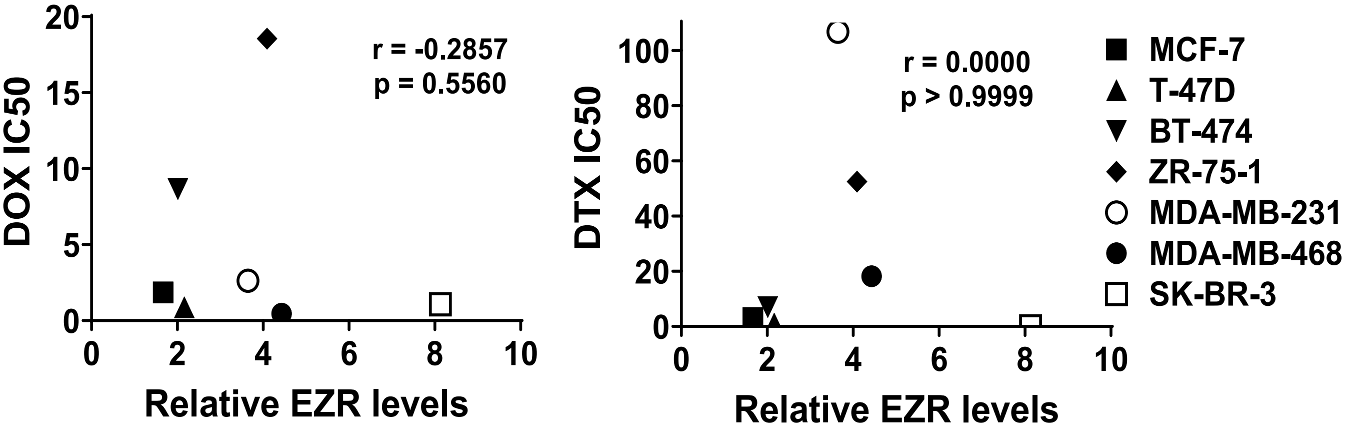

Supplementary Figure 1. Correlation analysis of ezrin levels with DOX or DTX sensitivity

(**A**) A summary of IC_{50} values ± standard deviations (SD) for DOX or DTX treatment for the BC cell lines listed. IC_{50} values were generated from cell viability analyses as described in Materials and Methods. Data shown are representative of 3 independent experiments. (**B**) Spearman's correlation analysis was performed between relative ezrin (EZR) protein levels as determined in Figure 1A and either DOX IC50 or DTX IC50, for the indicated cell lines.
