# Supplementary material for: Targeting the Ezrin Adaptor Protein Sensitizes Metastatic Breast Cancer Cells to Chemotherapy and Reduces Neoadjuvant Therapy–induced Metastasis
Source: Cancer Res Commun. 2022 Jun 17;2(6):456–70. doi: 10.1158/2767-9764.CRC-21-0117 (PMC10010290; doi:10.1158/2767-9764.CRC-21-0117)
Supplement: Figure S2 — Quantitation of exogenous Radixin and Moesin levels or knockdown by siRNA [file crc-21-0117-s05.pdf]

Supplementary Figure 2

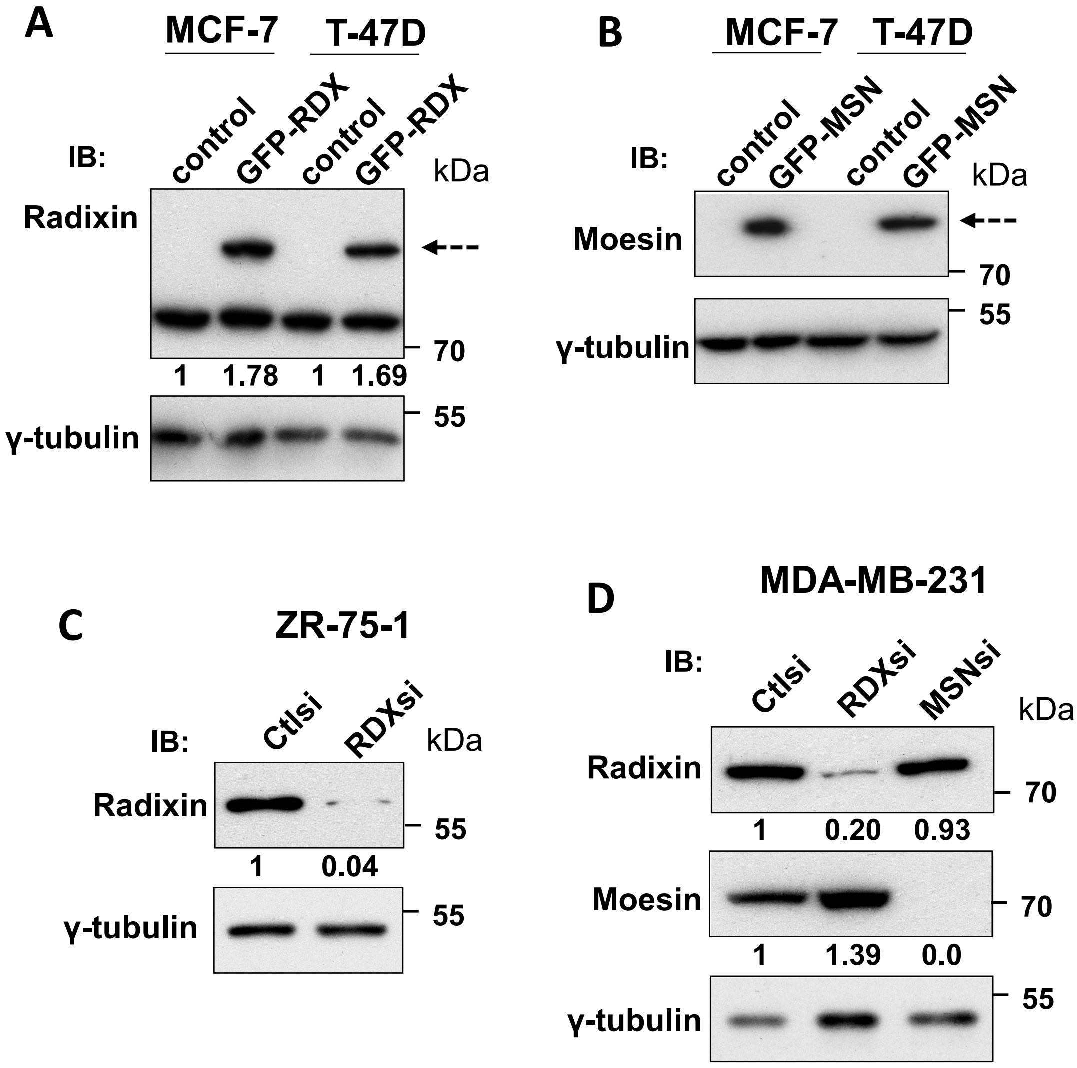

Supplementary Figure 2. Quantitation of exogenous Radixin and Moesin levels or knockdown by siRNA

(**A, B**) Immunoblotting analysis of radixin and moesin in MCF-7 and T-47D cells expressing GFP-radixin (RDX) or GFP-moesin (MSN). Exogenous protein is indicated by the dotted arrow. Tubulin is shown as a loading control. Quantitation of radixin over-expression by densitometry is shown under the indicated immunoblot. Moesin over-expression was not quantified as MCF-7 and T-47D cells do not endogenously express this protein. (**C, D**) Immunoblotting analysis of radixin and/or moesin knockdown in ZR-75-1 or MDA-MB-231 cells. Tubulin is shown as a loading control. Quantitation of radixin and/or moesin knockdown by densitometry is shown under the indicated immunoblots. Moesin is not expressed in ZR-75-1 cells and was not treated with moesin siRNA. Images shown are representative of 3 independent experiments.
