# Supplementary material for: Targeting the Ezrin Adaptor Protein Sensitizes Metastatic Breast Cancer Cells to Chemotherapy and Reduces Neoadjuvant Therapy–induced Metastasis
Source: Cancer Res Commun. 2022 Jun 17;2(6):456–70. doi: 10.1158/2767-9764.CRC-21-0117 (PMC10010290; doi:10.1158/2767-9764.CRC-21-0117)
Supplement: Figure S3 — Automated analysis of pTERM staining in metastatic lesions and adjacent normal lung tissue [file crc-21-0117-s06.pdf]

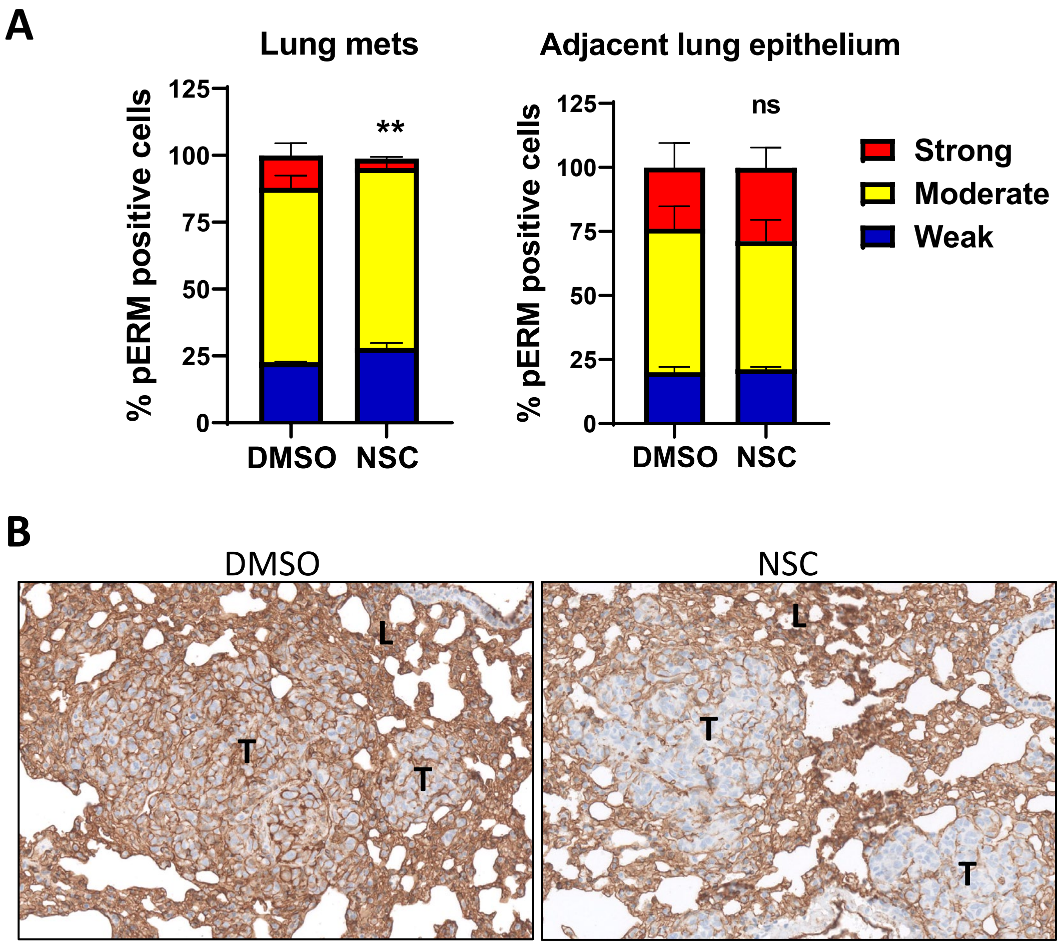

Supplementary Figure 3. Automated analysis of pTERM staining in metastatic lesions and adjacent normal lung tissue

Analysis of pTERM IHC staining was performed on FFPE control and ezrin inhibitor treated (NSC) lungs harvested from the experimental metastasis treatment study. Stained slides were scanned and digital images were analyzed by Halo^{TM} (Indica Labs). (**A**)The percentage of weak, moderate and strong staining cells within the lung metastases (left) and surrounding lung epithelium (right) was quantified. (**B**) Representative IHC images are shown with lung parenchyma (L) and tumor metastasis (T) indicated. N=4 per group. **p=0.0062 between DMSO and NSC % strong staining cells by two-way ANOVA with Sidak's post test.
