# Supplementary material for: Targeting the Ezrin Adaptor Protein Sensitizes Metastatic Breast Cancer Cells to Chemotherapy and Reduces Neoadjuvant Therapy–induced Metastasis
Source: Cancer Res Commun. 2022 Jun 17;2(6):456–70. doi: 10.1158/2767-9764.CRC-21-0117 (PMC10010290; doi:10.1158/2767-9764.CRC-21-0117)
Supplement: Figure S4 — Assessment of microscopic metastasis and lung metastatic burden [file crc-21-0117-s07.pdf]

Supplementary Figure 4

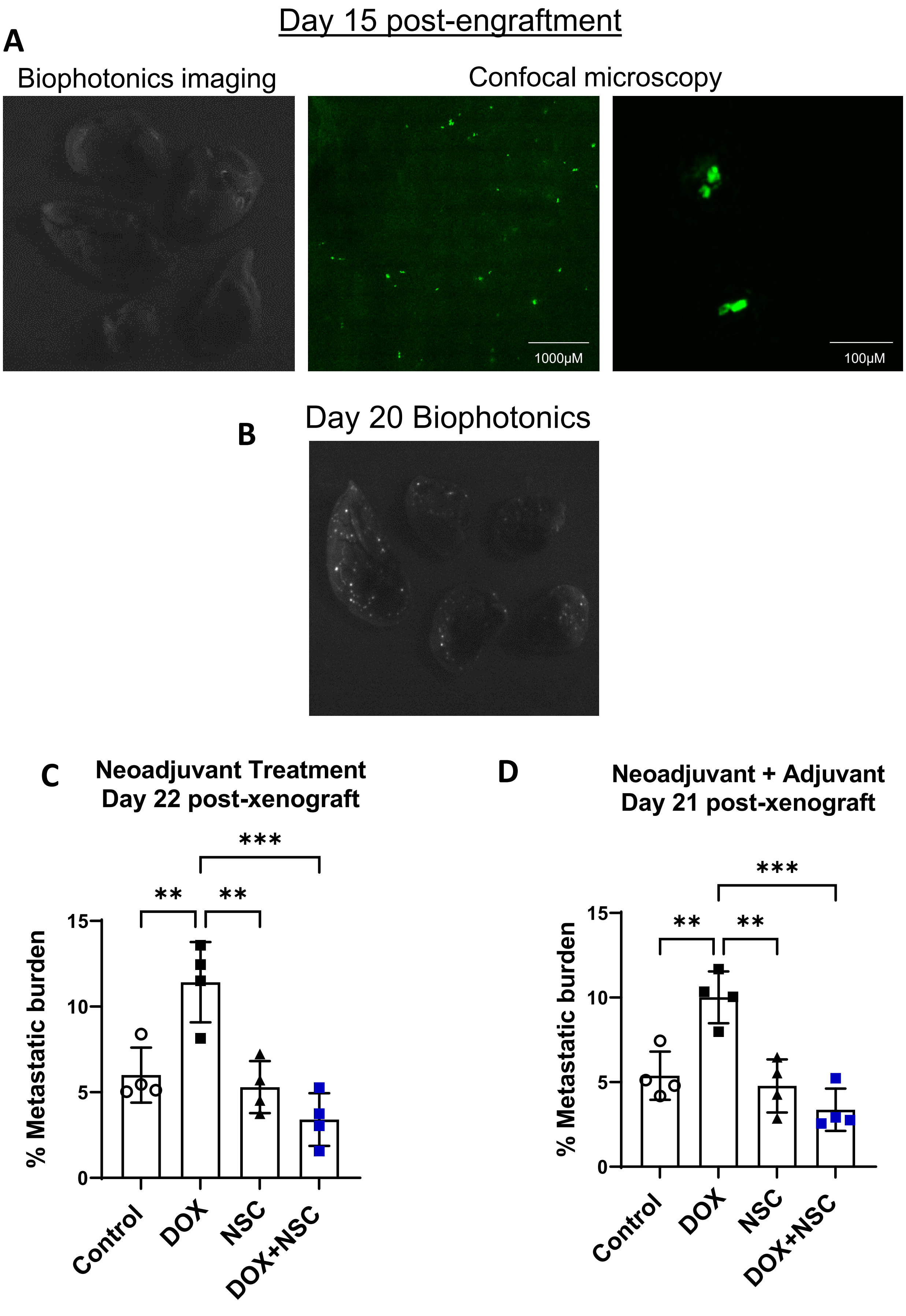

Supplementary Figure 4. Assessment of microscopic metastasis and lung metastatic burden

(**A, B**) Biophotonic images of lungs harvested from mice at day 15 (A, left panel) and day 20 (**B**) post-mammary fatpad injection were taken to determine the presence of metastasis. Metastases are not visible by this method until ~ day 20. Spinning Disk Confocal microscopy of lungs at day 15 post-engraftment was performed to confirm the presence of microscopic metastasis (Panel A, centre, right). (**C**) Metastatic burden at the time of tumor resection (~day 22) was assessed by biophotonic imaging and quantified as described in Materials and Methods. N=4 mice per group. (**D**) Assessment of metastatic burden day 21 post-engraftment in the neoadjuvant plus adjuvant treatment model by biophotonic imaging. N=4 mice per group. **$p<0.01$; ***$p<0.001$ using one-way ANOVA with Tukey's post test.
