# Supplementary material for: Targeting the Ezrin Adaptor Protein Sensitizes Metastatic Breast Cancer Cells to Chemotherapy and Reduces Neoadjuvant Therapy–induced Metastasis
Source: Cancer Res Commun. 2022 Jun 17;2(6):456–70. doi: 10.1158/2767-9764.CRC-21-0117 (PMC10010290; doi:10.1158/2767-9764.CRC-21-0117)
Supplement: Figure S5 — Anti-ezrin treatment does not alter levels of circulating tumor cells in vivo [file crc-21-0117-s08.pdf]

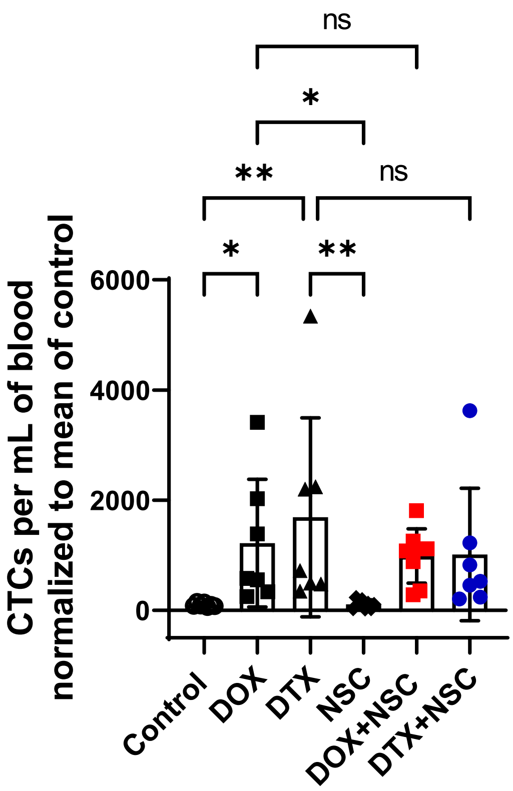

Supplementary Figure 5. Anti-ezrin treatment does not alter levels of circulating tumor cells *in vivo*

Peripheral blood was isolated, processed and analyzed for the presence of CTCs by flow cytometry for each treatment group as described in Materials and Methods. The number of CTCs per millilitre of blood was calculated and normalized to the mean of the control group in order to allow pooling of data from different experiments. Due to the large variation in CTCs detected within each group, p-values were calculated using one-way ANOVA with Kruskal-Wallis' post test. * p = 0.0176; ** p = 0.0066. N=7-8 per group.
