# Supplementary material for: Targeting the Ezrin Adaptor Protein Sensitizes Metastatic Breast Cancer Cells to Chemotherapy and Reduces Neoadjuvant Therapy–induced Metastasis
Source: Cancer Res Commun. 2022 Jun 17;2(6):456–70. doi: 10.1158/2767-9764.CRC-21-0117 (PMC10010290; doi:10.1158/2767-9764.CRC-21-0117)
Supplement: Figure S6 — Ezrin protein expression after DOX and DTX drug challenge [file crc-21-0117-s09.pdf]

Supplementary Figure 6

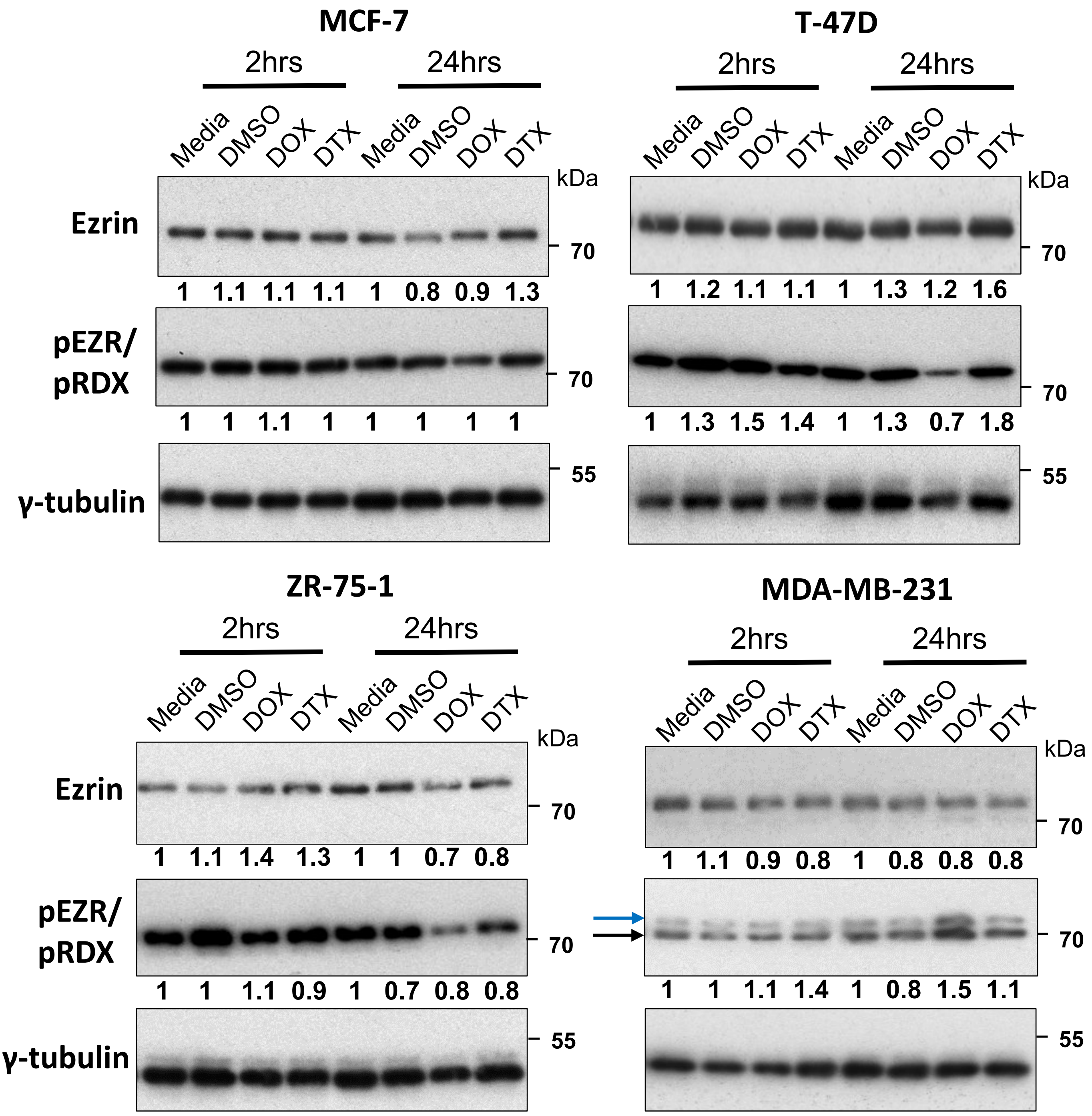

Supplementary Figure 6. Ezrin protein expression after DOX and DTX drug challenge

Immunoblotting analysis of ezrin in MCF-7, T-47D, ZR-75-1 and MDA-MB-231 cells after short term exposure to DOX or DTX. Cells were treated at for 2h or 24h based on the IC_{50} values of DOX/DTX for each cell line as determined in Supplementary Figure 1A. γ-tubulin is shown as a loading control. Densitometric analysis was performed and the relative ezrin levels for each treatment are shown. The values were first normalized to γ-tubulin and then made relative to the media only (media) control for each time point. Data are representative of 3 independent experiments. No statistically significant difference among the treatments was observed, based on one-way ANOVA with Dunnett's post test.
